# Ethylene‐Activated E3 Ubiquitin Ligase MdEAEL1 Promotes Apple Fruit Softening by Facilitating the Dissociation of Transcriptional Repressor Complexes

**DOI:** 10.1002/advs.202417393

**Published:** 2025-04-09

**Authors:** Tong Li, Li Liu, Guangxin Yang, Yingcong Cai, Yingda Wang, Bowen Sun, Le Sun, Weiting Liu, Aide Wang

**Affiliations:** ^1^ Key Laboratory of Fruit Postharvest Biology (Liaoning Province) Key Laboratory of Protected Horticulture (Ministry of Education) National & Local Joint Engineering Research Center of Northern Horticultural Facilities Design & Application Technology (Liaoning) College of Horticulture Shenyang Agricultural University Shenyang 110866 China; ^2^ Liaoning Institute of Pomology Xiongyue 115009 China

**Keywords:** apple fruit, ethylene, fruit softening

## Abstract

Fruit of most apple varieties soften after harvest, and although the hormone ethylene is known to induce softening, the associated pathway is not well resolved. In this study, it is determined that *MdEAEL1* (Ethylene‐activated E3 ubiquitin Like 1) is specifically expressed during apple fruit postharvest storage, activated by ethylene, and interacts with the transcription factor MdZFP3 (zinc finger protein3). MdZFP3 is found to rely on an EAR (ethylene‐responsive element binding factor‐associated amphiphilic repression) motif to form a transcriptional repression complex with MdTPL4 (TOPLESS4)‐MdHDA19 (histone deacetylase19), thereby downregulating the histone acetylation levels of the promoters of a range of cell wall degradation‐related genes and inhibiting their transcription. MdEAEL1 ubiquitinates and degrades MdZFP3, leading to the disassembly of the MdZFP3‐MdTPL4‐MdHDA19 transcriptional repression complex. This process promotes the transcription of cell wall degradation‐related genes, resulting in fruit softening during storage. Furthermore, the disassembly of the MdZFP3‐MdTPL4‐MdHDA19 transcriptional repression complex, mediated by MdEAEL1, upregulates the transcription of *MdEAEL1* itself, creating a feedback loop that further promotes softening. This study elucidates the interplay between post‐translational modifications of a transcription factor and its epigenetic modification to regulate fruit softening, and highlights the complexity of ethylene‐induced softening.

## Graphical abstract

Ethylene‐activated MdEAEL1 mediates the disassembly of the MdZFP3‐MdTPL4‐MdHDA19 transcriptional repression complex, upregulating the histone acetylation levels in the promoter regions of cell wall degradation‐related genes, consequently promoting softening during storage. This study elucidates the interplay between post‐translational modifications of a transcription factor and its epigenetic modification to regulate fruit softening, and highlights the complexity of ethylene‐induced softening.

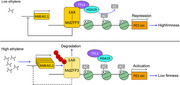

## 1 Introduction

Fruit softening is a highly complex phenomenon associated with the disassembly of the primary cell wall,^[^ ^1^ ^]^ which is predominantly composed of cellulose, hemicelluloses, and pectins, through the action of various cell wall modifying proteins. For example, the degradation of pectin is a prominent feature of cell wall degradation, as well as breakdown of the middle lamella.^[^ ^2^ ^]^ The proteins that contribute to ripening‐associated wall modification include polygalacturonase (PG), pectin methylesterase (PME), β‐galactosidase (β‐Gal), pectate lyase (PL), α‐arabinofuranosidase (α‐AFase), xyloglucan endotransglucosylase hydrolase (XET), and expansin (EXP) families.^[^ ^3^ ^]^ Tissue softening is a prominent characteristic of fruit senescence, leading to shortened shelf life, increased susceptibility to mechanical damage and microbial infection, diminished edibility, decreased commercial value, and higher transportation costs.^[^ ^4^ ^]^ Accordingly, understanding the mechanisms regulating these genes may provide valuable insights into both the fruit ripening process and the development of strategies to improve fruit quality and postharvest shelf life.

Apple (*Malus domestica*) is a typical respiratory climacteric fruit and during postharvest storage, an ethylene peak is produced as the respiration rate changes. The amount of ethylene generated during apple storage correlates with the rate of fruit senescence^[^ ^5^ ^]^ and treatment with the ethylene signaling inhibitor 1‐methylcyclopropene (1‐MCP) has the opposite effect.^[^ ^6^ ^]^ During softening, the expression of cell wall degradation‐related genes such as *PG*, *PL*, *β‐Gal*, *α‐AFase*, *XET*, and *EXP* is induced by ethylene.^[^ ^7^ ^]^ Transgenic experiments have found that overexpression of *MdPG1* promotes the degradation of middle lamella pectin in apple fruit, increasing the content of water‐soluble pectin, thereby promoting post‐harvest softening of apple fruit.^[^ ^8^ ^]^ Conversely, silencing *MdPG1* inhibits cell wall rupture and reduces the content of water‐soluble pectin, thereby suppressing post‐harvest softening of apple fruit.^[^ ^9^ ^]^ Therefore, *MdPG1* is identified as a key marker gene for apple fruit softening.^[^ ^10^ ^]^ Previous studies of apple have shown that the transcription factors MdEIL2 and MdCBF2 directly regulate the transcription levels of the *MdPG1*, and together, they regulate ethylene‐induced apple fruit softening during low‐temperature storage^[^ ^10^ ^]^ and that the transcription factors *MdMADS6*, *MdMADS8*, and *MdMADS9* directly regulate *MdPG1* transcription.^[^ ^11^ ^]^ In addition to transcriptional regulation, post‐translational modification of transcription factors, such as phosphorylation and ubiquitination, has also been found to play a role in ethylene‐induced fruit softening.^[^ ^12^ ^]^ For example, we recently found that ethylene‐induced MdPUB24 facilitates the ubiquitination and degradation of the transcription factor MdNAC72. Furthermore, this process can be enhanced by the ethylene‐induced phosphorylation of MdNAC72 by MdMAPK3, which weakens the transcriptional inhibition of MdNAC72 of *MdPG1*, thereby promoting fruit softening during storage.^[^ ^7^ ^]^ Previous studies have also shown that epigenetic mechanisms play a regulatory role in softening.^[^ ^13^ ^]^ For example, in tomato (*Solanum lycopersicum*) fruit, the ethylene‐repressed SlERF.F12 interacts with the co‐repressor TPLESS 2 (SlTPL2) through a C‐terminal EAR (ethylene‐responsive element binding factor‐associated amphiphilic repression) motif (LxLxLx or DLNxxP) and recruits the histone deacetylases SlHDA1 (histone deacetylase1) and SlHDA2 to form a trimer.^[^ ^14^ ^]^ This complex decreases the acetylation level of the promoter regions of cell wall disassembly‐related genes such as *SlPG2a* and *SlPL*, leading to the inhibition of gene expression and suppressed fruit softening. The EAR motif, is a prominent repression motif in transcription factors and interacts with the co‐repressors TPL (TOPLESS) and SAP18 (Sin3A‐associated Protein 18). This interaction recruits histone deacetylases to form an EAR‐mediated transcriptional repression complex (EAR‐TPL‐HDA or EAR‐SAP18‐HDA), thereby reducing histone acetylation levels in the promoter regions of downstream genes, thereby repressing their transcription.^[^ ^15^ ^]^ This transcription factor‐mediated epigenetic regulatory mechanism plays an important role in plant growth and development, including fruit ripening and senescence.^[^ ^16^ ^]^ The gene regulatory network that controls fruit softening is clearly complex, but it is not known whether there is coordinated regulation post‐translational modifications of transcription factors and transcription factor‐mediated epigenetic modifications.

Numerous types of zinc finger proteins (ZFPs) interact in plants with DNA, RNA, and other proteins, and play key roles in their cellular functions.^[^ ^17^ ^]^ The classes of ZFPs are based on the arrangement of cysteine and histidine residues in the zinc‐finger motif, including C2H2, C2C2, C2HC, C2C2C2C2, and C2HCC2C2.^[^ ^18^ ^]^ Of these, the C2H2 type has been extensively studied and represents one of the most prevalent ZFPs in eukaryotes; moreover, it serves as a transcription factor in plants and plays a role in DNA binding and transcription regulation.^[^ ^19^ ^]^ C2H2 ZFPs regulate the expression of genes related to plant growth and development under normal growth conditions and stress responses.^[^ ^20^ ^]^ In addition, two C2H2‐type ZFP transcription factors, MaC2H2‐1/2, have been identified in banana (*Musa acuminate*) that act as transcription activators that directly bind to the promoters of starch degradation‐related (*MaBAM4*, *MaBAM6*, *MaISA2*, and *MaPWD1*) and cell wall degradation‐related (*MaEXP‐A2*, *MaEXP‐A8*, and *MaSUR14*) genes, thereby promoting their transcription and post‐harvest ripening.^[^ ^21^ ^]^ However, it is not known how ZFP regulates the expression of its target genes and the postharvest fruit ripening and senescence, or whether post‐translational modifications of ZFP are involved in this process.

Here, we found that MdZFP3 recruits the co‐repressor MdTPL4 and the chromatin modifier protein HDA19 to form the transcriptional repression complex (MdZFP3‐MdTPL4‐MdHDA19), thereby epigenetically repressing the expression of cell wall degradation‐related genes. Furthermore, Ethylene‐Activated E3 ubiquitin Ligase 1 (MdEAEL1) mediates the ubiquitination and degradation of MdZFP3, leading to the dissociation of the MdZFP3‐MdTPL4‐MdHDA19 transcriptional repression complex. This results in the up‐regulation of histone acetylation levels in the promoters of cell wall degradation‐related genes, thereby promoting their transcription and accelerating softening during fruit storage.

## 2 Results

### 2.1 Ethylene Promotes Apple Fruit Softening and *MdEAEL1* Expression during Storage

**Figure 1:**
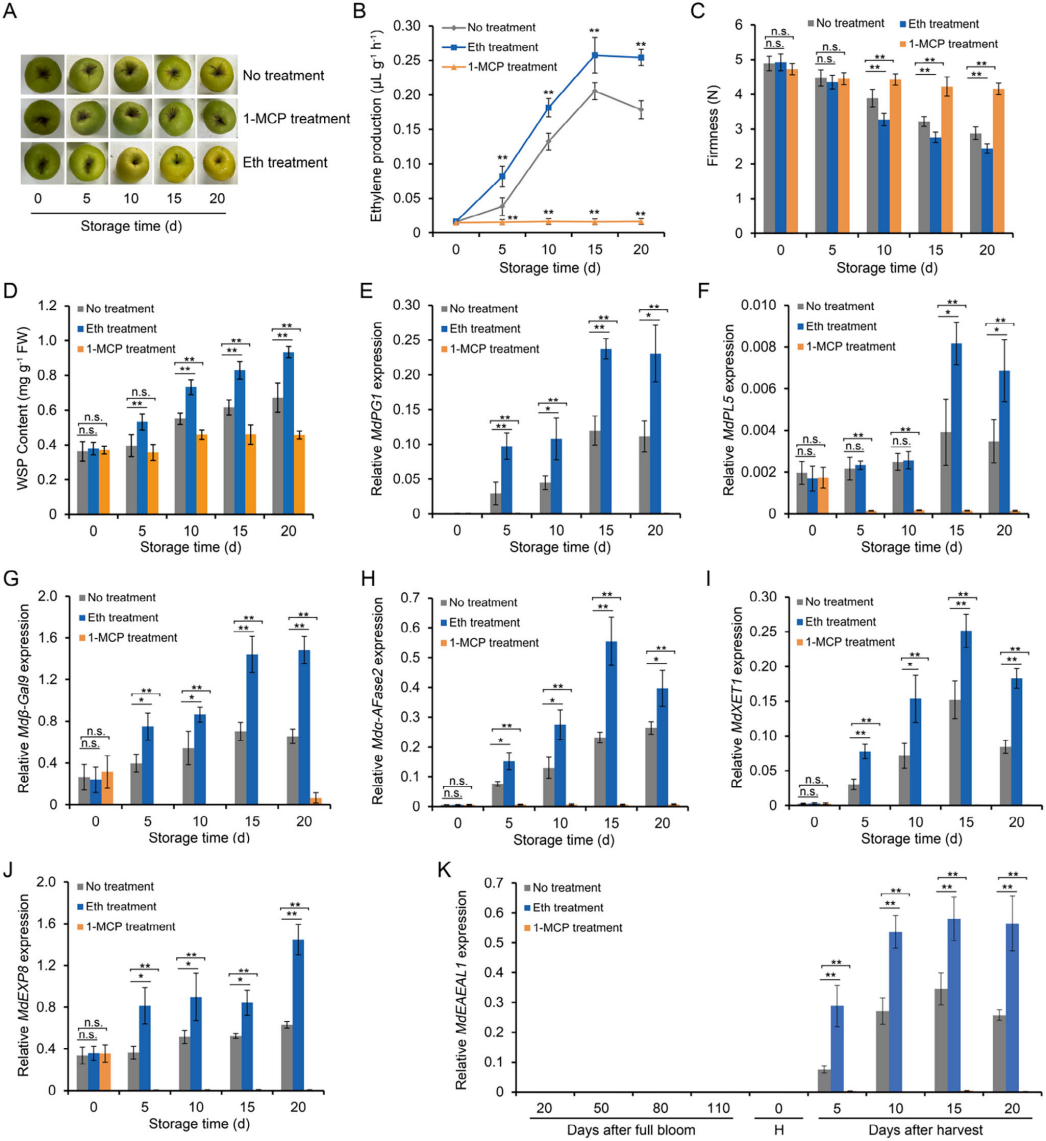
Ethylene promotes apple fruit softening and *MdEAEL1* expression during storage. Apple fruits were harvested 140 d after full bloom and treated with ethylene (ethephon) and 1‐MCP (ethylene signaling inhibitor), or not treated. A) Changes in the appearance of apples during the 20 d storage period. B) Ethylene production, C) firmness, and D) water‐soluble pectin (WSP) were measured. FW, Fresh weight. The data are presented as means ± SE (*n* = 5 groups, 10 fruits per group). Statistical significance was assessed using Student's *t*‐test (**p < 0.01). Expression of E) *MdPG1*, F) *MdPL5*, G) *Mdβ‐Gal9*, H) *Mdα‐AFase2*, I) *MdXET1*, J) *MdEXP8*, and K) *MdEAEL1* detected using reverse transcription‐quantitative PCR (RT‐qPCR). In (K), H is the harvest day (140 d after full bloom). The data are presented as means ± SE (*n* = 3 groups, 10 fruits per group). Statistical significance was assessed using Student's *t*‐test (**p < 0.01, *p < 0.05).

In a foundational experiment with stored apple fruit, we observed that the occurrence of an ethylene peak was accompanied by fruit softening, and that ethylene treatment promoted endogenous ethylene production, water‐soluble pectin (WSP) content and softening, whereas an ethylene signaling inhibitor, 1‐MCP, inhibited both (**Figure** **1**A–D). To investigate how ethylene triggers apple softening, we reanalyzed transcriptome data^[^ ^22^ ^]^ from three different groups of fruit: 0‐fruit (unstored), 15‐fruit (stored for 15 d), and 15‐MCP‐fruit (stored for 15 d after 1‐MCP treatment). We noted that six cell wall degradation‐related genes (*MdPG1*, *MdPL5*, *Mdβ‐Gal9*, *Mdα‐AFase2*, *MdXET1*, and *MdEXP8*) were expressed at higher levels in 15‐fruit compared to 0‐fruit but at lower levels in 15‐MCP‐fruit compared to 15‐fruit (Table S1, Supporting Information). These results were further confirmed using reverse transcription‐quantitative PCR (RT‐qPCR) (Figure 1E–J), suggesting a relationship between ethylene, the expression of these genes during fruit storage and softening. We also identified an E3 ubiquitin ligase gene in the transcriptome sequencing data that showed high expression after 15 d of storage but minimal expression after 1‐MCP treatment (Table S1, Supporting Information). RT‐qPCR of expression during fruit development and storage revealed that it was specifically expressed during storage stage and upregulated by ethylene treatment, while expression was completely inhibited by 1‐MCP treatment (Figure 1K). Accordingly, we named it *Ethylene‐activated E3 ubiquitin Ligase* ***1*** (*MdEAEL1*).

### 2.2 MdEAEL1 Promoted Softening of Apple Fruit during Storage

**Figure 2:**
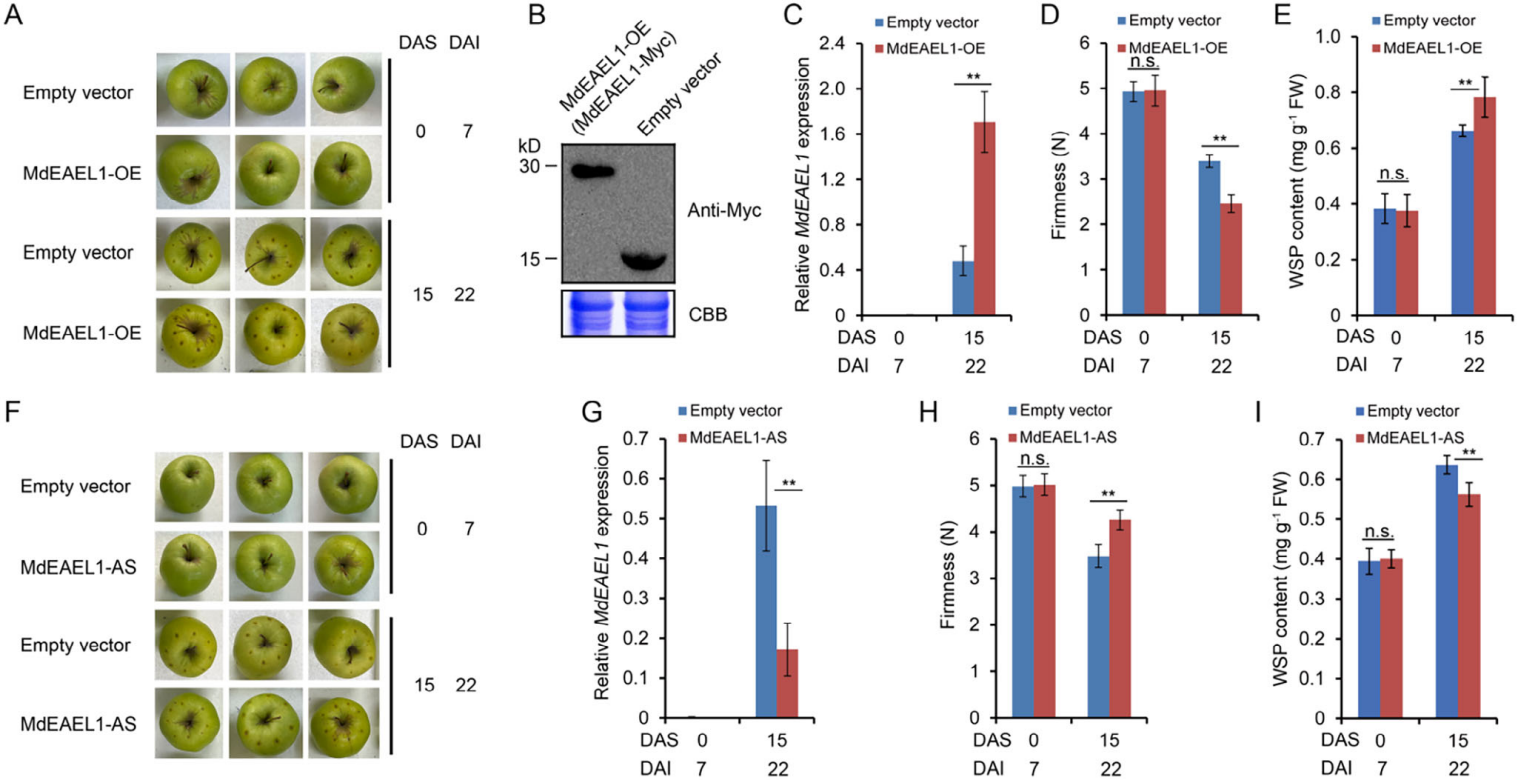
*MdEAEL1* promotes apple fruit softening during storage. A) Apple fruit transiently overexpressing *MdEAEL1* (*MdEAEL1*‐OE) or empty vector (pRI101‐Myc) during storage. *MdEAEL1*‐OE fruit were harvested 7 d after injection and stored at room temperature for 15 d. B) Proteins were extracted from apple fruit at the injection site. Immunoblot analysis was conducted using an anti‐Myc antibody for detection, with Coomassie brilliant blue (CBB) staining of protein extracts serving as a loading control. C) Reverse transcription‐quantitative PCR (RT‐qPCR) was used to detect the expression of *MdEAEL1* in *MdEAEL1*‐OE fruit and the empty vector fruit. D) Fruit firmness and E) water‐soluble pectin (WSP) were measured. FW, Fresh weight. F) Fruit transiently silenced *MdEAEL1* (*MdEAEL1*‐AS), with an empty vector as a control. *MdEAEL1*‐AS fruit were harvested 7 d after injection and stored at room temperature for 15 d. G) RT‐qPCR was used to detect the expression of *MdEAEL1* in the fruit of *MdEAEL1*‐AS and Empty vector. H) Fruit firmness and I) water‐soluble pectin (WSP) were measured. FW, Fresh weight. DAI, days after infiltration; DAS, days after storage. For firmness and WSP determination, the data are presented as means ± SE (*n* = 5 groups, 5 fruits per group). For RT‐qPCR, the data are presented as means ± SE (*n* = 3 groups, 10 fruits per group). Statistical significance was assessed using Student's *t*‐test (**p < 0.01, *p < 0.05).

To investigate the potential role of *MdEAEL1* in fruit metabolism during storage, it was transiently overexpressed in apples (*Malus domestica* cv. Golden Delicious; GD) using a *35S:Myc‐MdEAEL1* plasmid, while fruit infiltrated with the empty vector served as the control (**Figure** **2**A). We confirmed the accumulation of MdEAEL1 fused to a Myc tag by immunoblot analysis with an anti‐Myc antibody (Figure 2B). We found that *MdEAEL1* expression in fruit during storage was significantly higher in *MdEAEL1*‐overexpressing (*MdEAEL1*‐OE) fruit compared to control fruit (Figure 2C) and the *MdEAEL1*‐OE fruit softened faster than control fruit (Figure 2D). Furthermore, WSP content increased (Figure 2E) and genes related to cell wall degradation, such as *MdPG1*, *MdPL5*, *Mdβ‐Gal9*, *Mdα‐AFase2*, *MdXET1*, and *MdEXP8*, were upregulated in *MdEAEL1*‐OE fruits (Figure S1A–F, Supporting Information). Conversely, transient silencing of *MdEAEL1* (*MdEAEL1*‐AS) resulted in firmer fruit and decreased expression of the cell wall degradation‐related genes and WSP content (Figure 2F–I and Figure S1G–L, Supporting Information).

### 2.3 MdEAEL1 Interacted Directly with MdZFP3

**Figure 3:**
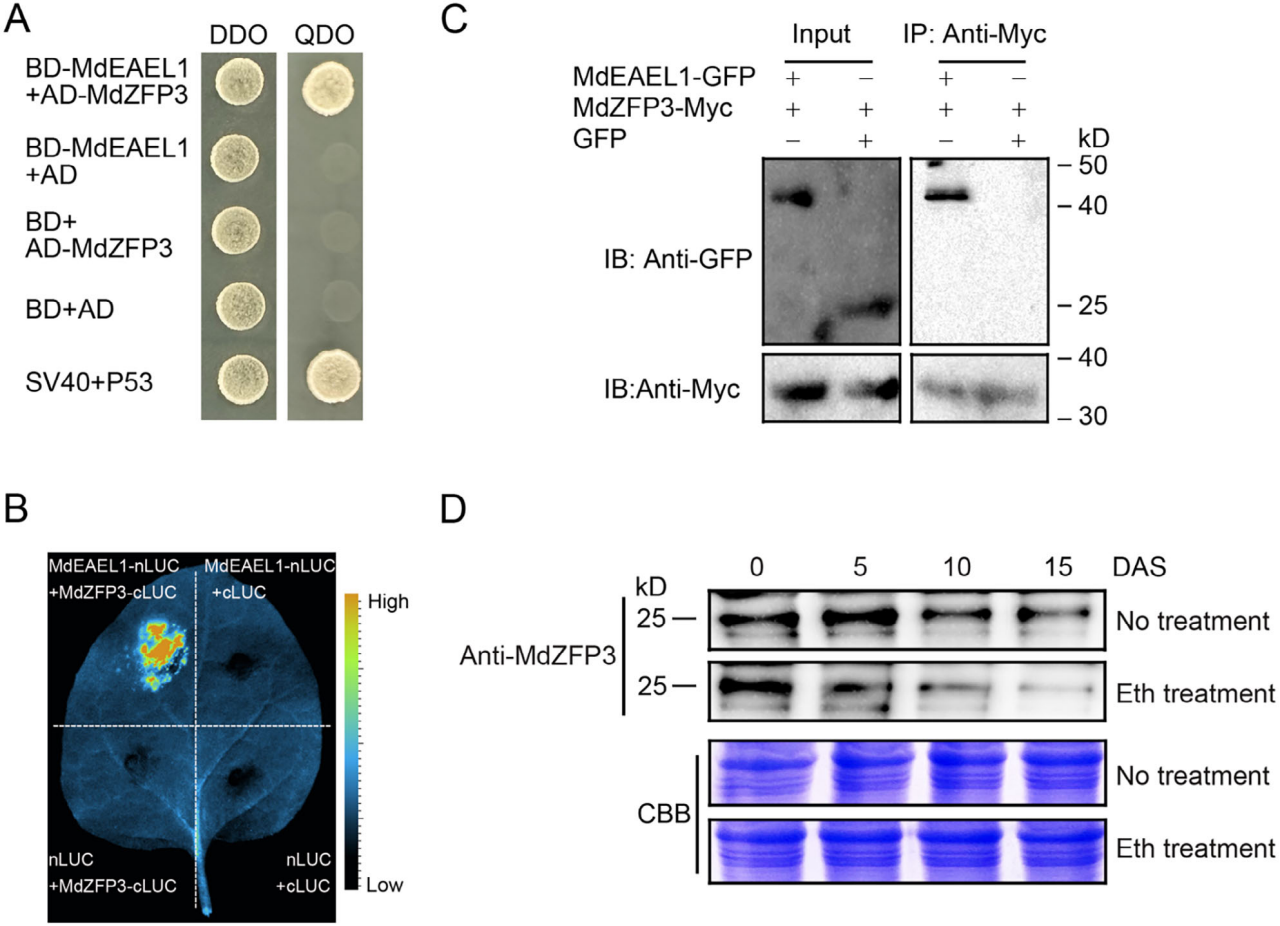
MdEAEL1 interaction with MdZFP3 and MdZFP3 expression. A) Interaction between MdEAEL1 and MdZFP3 in a yeast‐two‐hybrid (Y2H) assay. DDO, synthetic defined (SD) medium lacking Trp and Leu; QDO, SD medium lacking Trp, Leu, His, and Ade. Negative controls included BD (binding domain) and AD (activation domain) vectors, while the positive control used SV40/P53. B) Interaction between MdEAEL1 and MdZFP3 in *N. benthamiana* leaves via luciferase complementation imaging (LCI). C) Interaction between MdEAEL1 and MdZFP3 was verified using a co‐immunoprecipitation (co‐IP) assay. MdZFP3 tagged with Myc (MdZFP3‐Myc) and MdEAEL1 fused with green fluorescent protein (MdEAEL1‐GFP) were overexpressed in apple fruit calli, respectively. The immunoprecipitation analysis was conducted using an anti‐Myc antibody and immunoblotting was performed using anti‐GFP and anti‐Myc antibodies. D) Immunoblot analysis of MdZFP3 protein expression levels during apple fruit storage using an anti‐MdZFP3 antibody. Coomassie Brilliant Blue (CBB) staining of the protein extracts from apple fruit served as a control to ensure equal loading. DAS, days after storage.

To further investigate the role of ethylene‐activated *MdEAEL1* in promoting apple softening during storage, we screened an apple fruit yeast two‐hybrid library using MdEAEL1 as bait. From a total of 98 positive clones, we identified 15 different genes, one of which is predicted to encode MdZFP3 protein (Table S2, Supporting Information). This revealed the C2H2 ZFP transcription factor MdZFP3 as a potential interacting protein. Notably, a C2H2 ZFP transcription factor has been reported to play a role in regulating cell wall degradation‐related genes in fruits,^[^ ^21^, ^23^ ^]^ so we hypothesized that the interaction between MdEAEL1 and MdZFP3 is involved in apple fruit softening during storage. To confirm this, we conducted a yeast two‐hybrid (Y2H) assay to confirm the interaction between MdEAEL1 and MdZFP3. Y2H‐gold yeast cells co‐transformed with binding domain (BD) (pGBKT7)‐MdEAEL1 and activation domain (AD) (pGADT7)‐MdZFP3 plasmids grew normally on selective medium (synthetic defined (SD)/‐Trp‐Leu‐His‐Ade), while Y2H‐gold yeast cells simultaneously transformed with BD‐MdEAEL1 and AD or BD and AD‐MdZFP3 plasmids did not grow (**Figure** **3**A). In addition to this in vitro interaction analysis, we performed an in vivo luciferase complementation imaging (LCI) assay in *Nicotiana benthamiana* (*N. benthamiana*) leaves, which showed a strong luminescence signal upon co‐expression of MdEAEL1‐nLUC and MdZFP3‐cLUC, suggesting an interaction between MdEAEL1 and MdZFP3. Negative controls, such as MdEAEL1‐nLUC/cLUC, nLUC/MdZFP3‐cLUC, and nLUC/cLUC, did not exhibit luminescence signals (Figure 3B). Furthermore, a co‐immunoprecipitation (co‐IP) assay demonstrated the interaction between MdEAEL1 fused with green fluorescent protein (GFP) and MdZFP3 fused to Myc in apple calli. No interaction was detected when a GFP empty vector was used (Figure 3C). These results indicated that MdEAEL1 interacts with MdZFP3 in vivo.

An immunoblot analysis showed that the abundance of the MdZFP3 protein gradually decreased with an increase in ethylene production during fruit storage and that ethylene treatment accelerated the degradation rate of MdZFP3 protein (Figure 3D). This suggested that MdZFP3 responds to ethylene and undergoes degradation during fruit storage.

### 2.4 MdZFP3 Acts as a Transcriptional Repressor, Inhibiting Apple Fruit Softening during Storage

**Figure 4:**
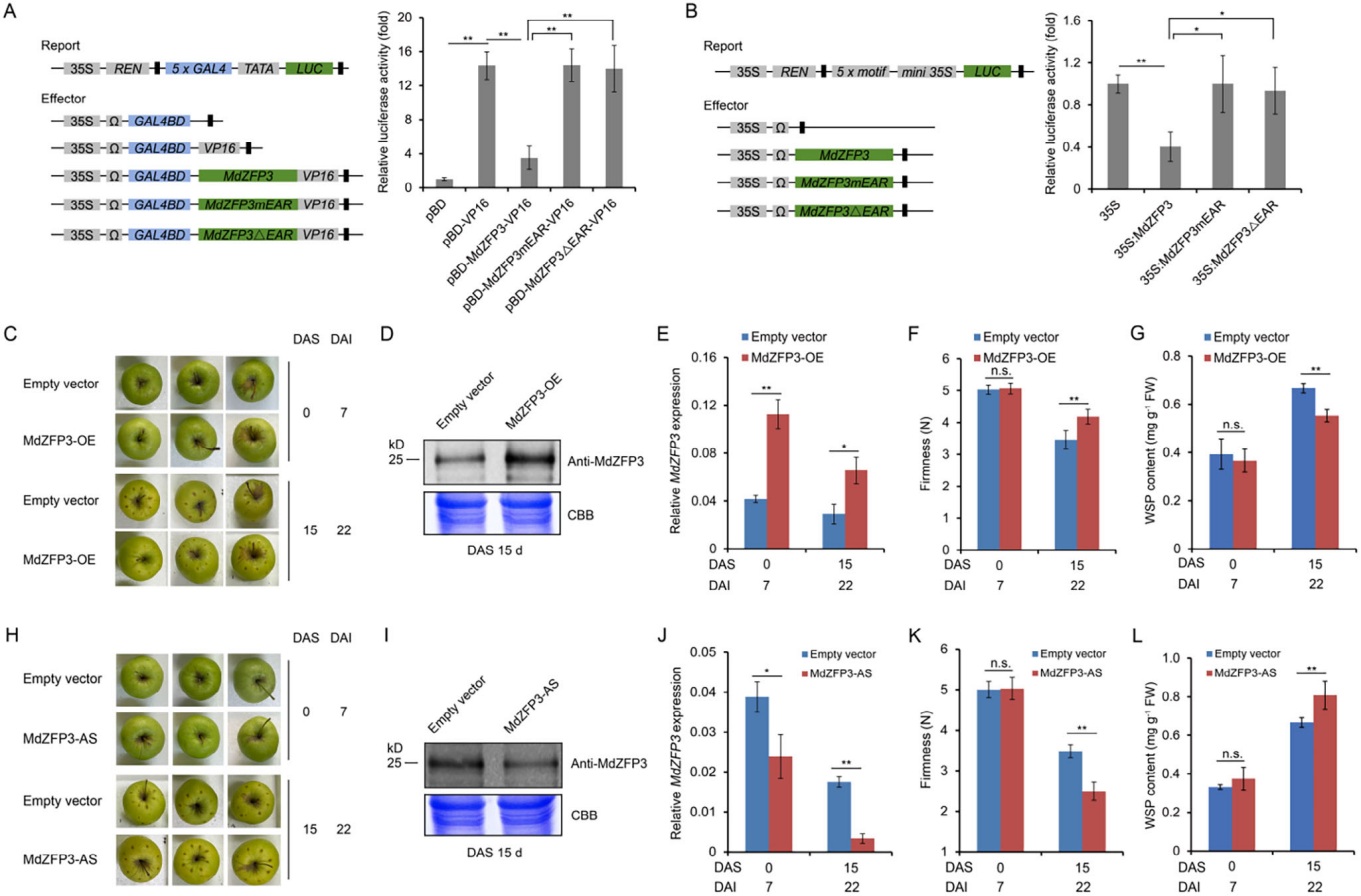
MdZFP3 is a transcriptional repressor that negatively regulates apple fruit softening. A) Transcriptional repression assay of MdZFP3. pBD, empty vector, negative control. pBD‐VP16, VP16 transcriptional activator domain, positive control. B) MdZFP3 functions as a transcriptional repressor of promoters containing ZFP transcription factor 5 × binding element. For A and B, the dual LUC/REN reporter was co‐transfected into *N. benthamiana* leaves along with individual effector plasmids. MdZFP3mEAR, the amino acids LGLDL in the EAR motif of MdZFP3 were mutated to SDSDS. MdZFP3△EAR, the EAR motif deleted from MdZFP3. The data are presented as means ± SE (*n* = 3 independent transfected *N. benthamiana* leaves). Statistical significance was determined using Student's *t*‐test (**p < 0.01, *p < 0.05). C) Apple fruit transiently overexpressing *MdZFP3* (*MdZFP3*‐OE) or empty vector (pRI101) during storage. *MdZFP3*‐OE fruit were harvested 7 d after injection and stored at room temperature for 15 d. D) Proteins were extracted from apple fruit (*MdZFP3*‐OE) at the injection site. Immunoblot analysis was conducted using an anti‐MdZFP3 antibody, with Coomassie brilliant blue (CBB) staining of protein extracts serving as a loading control. E) Reverse transcription‐quantitative PCR (RT‐qPCR) was used to detect the expression of *MdZFP3* in the fruits of *MdZFP3*‐OE and Empty vector. F) Fruit firmness and G) water‐soluble pectin (WSP) were measured. FW, Fresh weight. H) Apple fruit with transiently silenced *MdZFP3* expression (*MdZFP3*‐AS), with an empty vector as a control. *MdZFP3*‐AS fruit were harvested 7 d after injection and stored at room temperature for 15 d. I) Proteins were extracted from apple fruit (*MdZFP3*‐AS) at the injection site. Immunoblot analysis with an anti‐MdZFP3 antibody for detection, and Coomassie brilliant blue (CBB) staining of protein extracts serving as a loading control. J) RT‐qPCR analysis of the expression of *MdZFP3* in the fruits of *MdZFP3*‐AS and Empty vector. K) Fruit firmness and L) water‐soluble pectin (WSP) were measured. FW, Fresh weight. DAI, days after infiltration; DAS, days after storage. The data statistical analysis was used as described in Figure 2.

The interaction between ethylene‐activated MdEAEL1 and MdZFP3 suggested that MdZFP3 may be associated with ethylene signaling and the regulation of fruit softening. An analysis of the MdZFP3 protein sequence revealed a canonical EAR motif in the C‐terminus (Figure S2, Supporting Information), consistent with MdZFP3 acting as a transcriptional repressor. To test this, we constructed three vectors: MdZFP3, MdZFP3mEAR (where the LxLxL motif in the EAR motif was mutated to SxSxS), and MdZFP3ΔEAR (with the EAR motif deleted), aiming to inhibit VP16 activator‐mediated transactivation from Herpes simplex in transient expression assays. MdZFP3 inhibited the luciferase (LUC) activity promoted by VP16, while MdZFP3‐mEAR and MdZFP3‐ΔEAR led to a loss of the repression ability of MdZFP3 (**Figure** **4**A), suggesting that the transcriptional inhibition of MdZFP3 is mostly dependent on C‐terminal EAR motif. We also constructed a reporter vector containing the binding element of MdZFP3 upstream of the LUC gene, within a plasmid that overexpresses Renilla luciferase (REN) as an internal control. Transient expression assays showed that MdZFP3 repressed the transcriptional activity of the LUC reporter, while MdZFP3mEAR and MdZFP3ΔEAR showed no repression (Figure 4B). These results indicated that MdZFP3 represses the transcription of promoters containing the MdZFP3 binding element in an EAR‐dependent manner. In addition, we found that the expression of *MdZFP3* gradually decreases during apple fruits storage (Figure S3, Supporting Information).

To further investigate the role of *MdZFP3* during apple fruit storage, the gene was transiently overexpressed in GD apples using a *35S:MdZFP3* plasmid, with fruit infiltrated with the empty vector serving as the control (Figure 4C). We confirmed the overexpression of MdZFP3 by immunoblot analysis with an anti‐MdZFP3 antibody (Figure 4D). In addition, we found that *MdZFP3* expression in fruit during storage was significantly higher in *MdZFP3*‐overexpression (*MdZFP3*‐OE) fruit compared to the control fruit (Figure 4E), and the *MdZFP3*‐OE fruit softened at a slower rate than the control fruit (Figure 4F). Furthermore, the expression of genes related to fruit cell wall degradation (*MdPG1*, *MdPL5*, *Mdβ‐Gal9*, *Mdα‐AFase2*, *MdXET1*, and *MdEXP8*) and WSP content was lower in *MdZFP3*‐OE fruit (Figure 4G and Figure S4A–F, Supporting Information). In contrast, transient silencing of *MdZFP3* (*MdZFP3*‐AS fruit) had the opposite effect (Figure 4H–L and Figure S4G–L, Supporting Information). These results suggested that MdZFP3 is an EAR motif‐dependent transcriptional repressor, inhibiting the expression of wall degradation‐related genes and consequently delaying apple fruit softening during storage.

### 2.5 MdZFP3 Directly Suppresses the Expression of Cell Wall Degradation‐Related Genes

**Figure 5:**
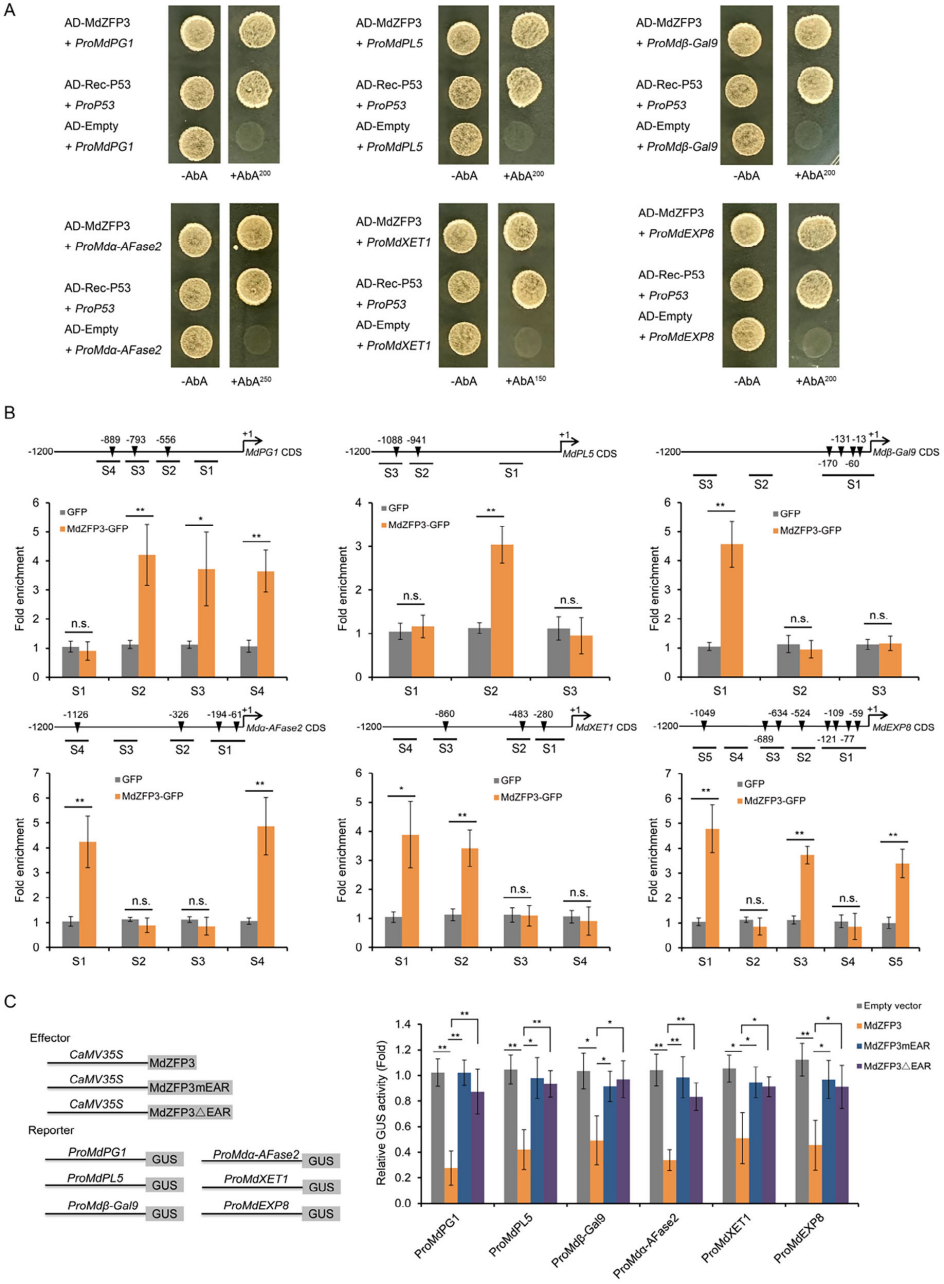
MdZFP3 inhibits the expression of cell wall degradation‐associated genes by binding to their promoters. A) Yeast one‐hybrid (Y1H) assay showed that MdZFP3 directly binds to the promoters of *MdPG1*, *MdPL5*, *Mdβ‐Gal9*, *Mdα‐AFase2*, *MdXET1*, and *MdEXP8*. For *ProMdPG1*, *ProMdPL5*, *ProMdβ‐Gal9*, and *ProMdEXP8*, the basal concentration of AbA (aureobasidin A) was 200 ng mL^−1^. For *ProMdα‐AFase2*, the concentration was 250 ng mL^−1^. For *ProMdXET1*, the concentration was 150 ng mL^−1^. B) Chromatin immunoprecipitation (ChIP)‐qPCR assay to assess the in vivo binding of MdZFP3 to the *MdPG1*, *MdPL5*, *Mdβ‐Gal9*, *Mdα‐AFase2*, *MdXET1*, and *MdEXP8* promoters. Chromatin samples from *35S:MdZFP3‐GFP* transgenic apple fruit calli were crosslinked and immunoprecipitated with an anti‐GFP antibody. The eluted DNA was used for qPCR amplification of sequences near the ZFP binding site, with different regions (S1–S4, S1–S3, or S1–S5) investigated. Negative control samples were obtained from *35S:GFP* (empty vector) transgenic fruit calli. The data are presented as means ± SE (*n* = 3 independent transgenic calli). Statistical significance was assessed using a Student's *t*‐test (**p < 0.01, *p < 0.05). C) GUS reporter gene assays showing that MdZFP3 depends on EAR motif to inhibit the expression of cell wall degradation‐related genes. The GUS reporter plasmid was co‐transfected into *N. benthamiana* leaves along with individual effector plasmids. MdZFP3mEAR, the amino acids LGLDL in the EAR motif of MdZFP3 were mutated to SDSDS. MdZFP3△EAR, EAR motif deleted from MdZFP3. The data are presented as means ± SE (*n* = 3 independent transfected *N. benthamiana* leaves). Statistical significance was determined using Student's *t*‐test (**p < 0.01, *p < 0.05).

The previous results showed that MdZFP3 inhibits the transcription of cell wall degradation‐related genes (*MdPG1*, *MdPL5*, *Mdβ‐Gal9*, *Mdα‐AFase2*, *MdXET1*, and *MdEXP8*). Notably, the binding motif A[AG/CT]CNAC of the ZFP transcription factor^[^ ^24^ ^]^ is present in the promoter regions of these genes (Figure S5, Supporting Information). Therefore, we hypothesized that MdZFP3 directly regulates their expression during apple fruit storage. A yeast one‐hybrid (Y1H) analysis showed that MdZFP3 directly binds to their promoter regions (**Figure** **5**A), and this was further confirmed through in vivo experiments using a chromatin immunoprecipitation (ChIP) assay with transgenic apple calli. MdZFP3 fused to GFP (*35S:GFP‐MdZFP3*) or GFP alone (*Pro35S:GFP*) were expressed in apple calli, then chromatin samples were extracted and incubated with an anti‐GFP antibody. The eluted DNA was used to amplify the sequences neighboring the A[AG/CT]CNAC motif using quantitative PCR (qPCR). This revealed that the S2/S3/S4 region of the *MdPG1* promoter, the S2 region of the *MdPL5* promoter, the S1 region of the *Mdβ‐Gal9* promoter, the S1/S4 region of the *Mdα‐AFase2* promoter, the S1/S2 region of the *MdXET1* promoter, and the S1/S3/S5 region of the *MdEXP8* promoter, each containing the A[AG/CT]CNAC motif, were highly enriched in the immunoprecipitated chromatin relative to the chromatin from the empty vector (*Pro35S:GFP*) calli (Figure 5B). This suggested that MdZFP3 directly binds to the *MdPG1*, *MdPL5*, *Mdβ‐Gal9*, *Mdα‐AFase2*, *MdXET1*, and *MdEXP8* promoters. Subsequently, we conducted a β‐glucuronidase (GUS) reporter gene assay in *N. benthamiana* leaves to determine the effects of MdZFP3, MdZFP3mEAR, and MdZFP3ΔEAR on the transcription of *MdPG1*, *MdPL5*, *Mdβ‐Gal9*, *Mdα‐AFase2*, *MdXET1*, and *MdEXP8*. When *35S:MdZFP3* was co‐transformed with the promoter regions of these genes (*ProMdPG1:GUS*, *ProMdPL5:GUS*, *ProMdβ‐Gal9:GUS*, *ProMdα‐AFase2:GUS*, *ProMdXET1:GUS*, and *ProMdEXP8:GUS*), the GUS signal was significantly reduced. Under the same conditions, the inhibitory effect on GUS activity was significantly less when *35S:MdZFP3mEAR* or *35S:MdZFP3ΔEAR* were expressed (Figure 5C).

### 2.6 MdZFP3 Interacts with MdTPL4 via the EAR Motif, Forming the MdZFP3‐MdTPL4‐MdHDA19 Complex to Repress the Transcription of Cell Wall Degradation‐Related Genes

**Figure 6:**
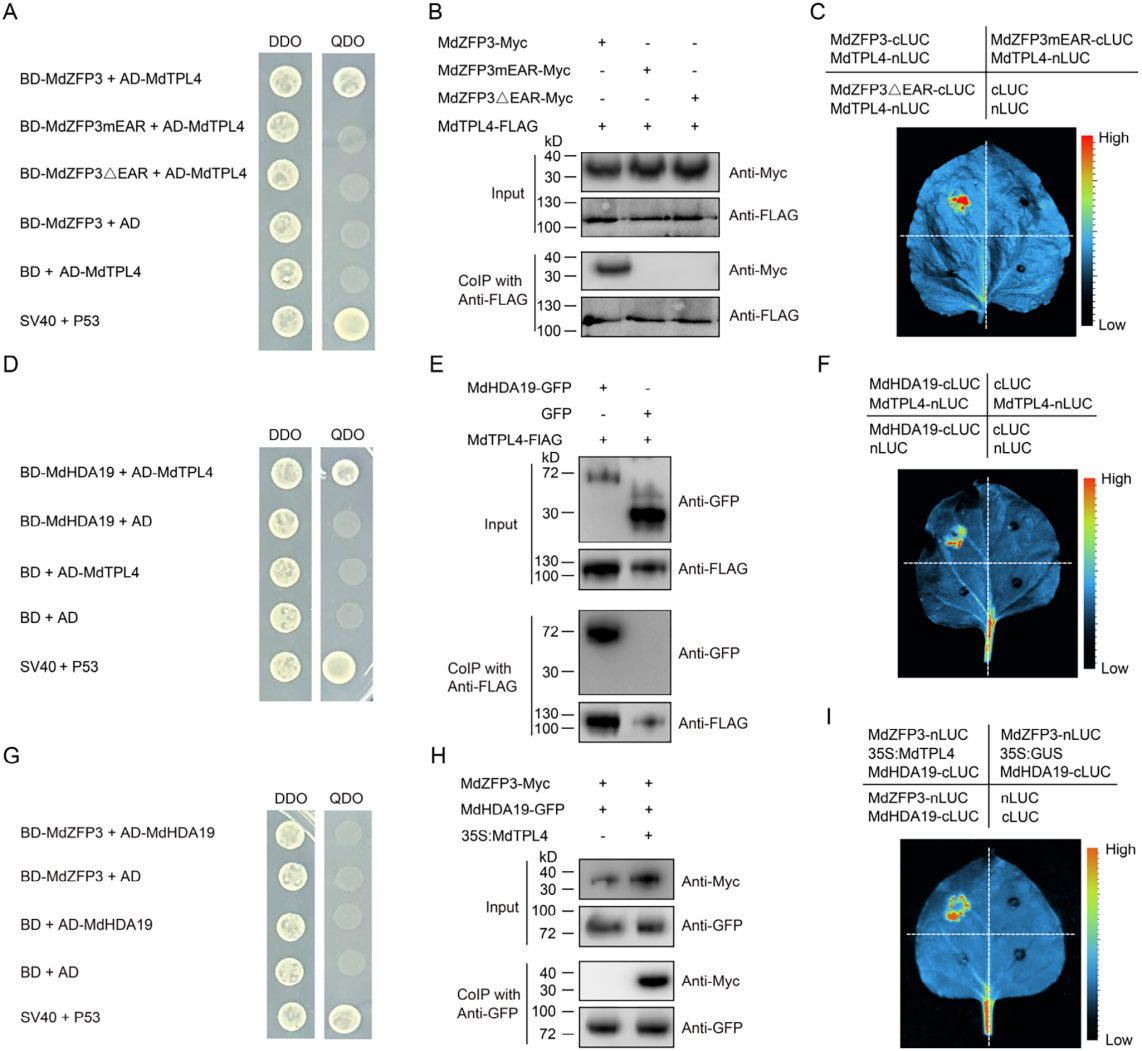
MdZFP3 interacts with MdTPL4 via the EAR motif to form the MdZFP3‐MdTPL4‐MdHDA19 complex. A) Yeast two‐hybrid (Y2H) assays showing that MdZFP3 interacts with MdTPL4, while MdZFP3mEAR, and MdZFP3△EAR do not interact with MdTPL4. B) The interaction relationship of MdTPL4 with MdZFP3, MdZFP3mEAR, or MdZFP3△EAR verified in co‐immunoprecipitation (co‐IP) assays. MdZFP3, MdZFP3mEAR, or MdZFP3△EAR tagged with Myc (MdZFP3‐Myc, MdZFP3mEAR‐Myc, or MdZFP3△EAR‐Myc) and MdTPL4 fused with FLAG (MdTPL4‐FLAG) were overexpressed in apple fruit calli. The immunoprecipitation analysis was conducted using an anti‐FLAG antibody and immunoblotting was performed using anti‐Myc and anti‐FLAG antibodies. C) The interaction relationship of MdTPL4 with MdZFP3, MdZFP3mEAR, or MdZFP3△EAR was confirmed in *N. benthamiana* leaves using luciferase complementation imaging (LCI). D) MdHDA19 interacts with MdTPL4 as shown in Y2H assays. E) The interaction of MdHDA19 with MdTPL4 was verified in a co‐IP assay. MdHDA19 tagged with GFP (MdHDA19‐GFP) and MdTPL4 fused with FLAG (MdTPL4‐FLAG) were overexpressed in apple fruit calli. The immunoprecipitation analysis was conducted using an anti‐FLAG antibody and immunoblotting was performed using anti‐GFP and anti‐FLAG antibodies. F) The interaction relationship MdHDA19 with MdTPL4 was confirmed in *N. benthamiana* leaves by LCI. G) MdHDA19 does not interact with MdTPL4, as shown in Y2H assays. The Y2H assay was performed as described in Figure 3. H) MdTPL4 mediates the interaction between MdZFP3 and MdHDA19 in a co‐IP assay. MdHDA19 tagged with GFP (MdHDA19‐GFP) and Myc‐fused MdZFP3 (MdZFP3‐Myc) were co‐expressed along with *35S:MdTPL4* in *N. benthamiana* leaves. As a control, only MdHDA19‐GFP and MdZFP3‐Myc were co‐expressed. The immunoprecipitation analysis was conducted using an anti‐GFP antibody and immunoblotting was performed using anti‐GFP and anti‐Myc antibodies. I) MdTPL4 mediates the interaction between MdZFP3 and MdHDA19 in *Nicotiana benthamiana* leaves via LCI assays. 35S:GUS, β‐glucuronidase protein is expressed in tobacco leaves as a negative control.

To elucidate the mechanism of transcriptional repression of target genes mediated by MdZFP3, we used MdZFP3 as bait to screen an apple fruit cDNA library in order to identify potential interacting proteins. From 21 positive clones, we identified 9 different genes, one of which encodes the TOPLESS protein (MdTPL4). Previous studies have shown that TOPLESS interacts with transcription factors containing the EAR motif.^[^ ^15^ ^]^ We validated the interaction between MdZFP3 and MdTPL4 using a Y2H assays, and observed that when the EAR motif was mutated (MdZFP3mEAR) or deleted (MdZFP3△EAR), this interaction was abolished (**Figure** **6**A). In addition, we performed a co‐IP assay in transgenic apple calli (*35S:Myc‐MdZFP3*/*35S:FLAG‐MdTPL4*, *35S:Myc‐MdZFP3mEAR*/*35S:FLAG‐MdTPL4*, or *35S:Myc‐MdZFP3△EAR*/*35S:FLAG‐MdTPL4*) to further validate the interaction between MdZFP3 and MdTPL4 *in planta*. We found that Myc‐tagged MdZFP3 interacts with MdTPL4 tagged with the FLAG epitope. We did not detect any interaction between the Myc‐tagged MdZFP3mEAR and MdZFP3△EAR with MdTPL4 (Figure 6B). Finally, we employed an LCI assay to validate this interaction. Constructs were created that fused MdTPL4 with the N‐terminus of luciferase (MdTPL4‐nLUC) and MdZFP3, MdZFP3mEAR, or MdZFP3△EAR with the C‐terminus of luciferase (MdZFP3‐cLUC, MdZFP3mEAR‐cLUC, or MdZFP3△EAR‐cLUC). These constructs were co‐infiltrated into the leaves of *N. benthamiana* and LUC signals were observed in the region co‐expressing MdTPL4‐nLUC and MdZFP3‐cLUC, but not MdTPL4‐nLUC and MdZFP3mEAR‐cLUC, MdTPL4‐nLUC and MdZFP3△EAR‐cLUC, or in the negative control (Figure 6C). These results collectively support an interaction between MdZFP3 and MdTPL4, both in vitro and *in plants*, that is dependent on the EAR motif.

Plant transcription factors containing an EAR motif have been shown to regulate downstream gene expression by recruiting TPL‐HDA19 protein complexes to alter deacetylation activity, including in ripening apple fruit.^[^ ^15^, ^16^ ^]^ We found evidence of an interaction between MdTPL4 and MdHDA19 using a Y2H assay (Figure 6D), a co‐IP experiment in transgenic apple calli transformed with *35S:GFP‐MdHDA19*/*35S:FLAG‐MdTPL4* or *35S:GFP*/*35S:FLAG‐MdTPL4* (Figure 6E) and an LCI assay (Figure 6F). Our previous results indicated that MdZFP3 interacts with MdTPL4, and MdTPL4 interacts with MdHDA19, suggesting that MdZFP3, MdTPL4, and MdHDA19 may form a trimer complex. However, a Y2H assay and a co‐IP experiment in the transient infection of *N. benthamiana* leaves (*35S:Myc‐MdZFP3*/*35S:GFP‐MdHDA19*) (Figure 6G,H lane 1) did not show an interaction between MdZFP3 and MdHDA19. Interestingly, co‐IP in the transient infection of *N. benthamiana* leaves (*35S:Myc‐MdZFP3*/*35S:GFP‐MdHDA19*/*35S:MdTPL4*) and LCI assays demonstrated that when MdTPL4 acts as an intermediate, MdZFP3 and MdHDA19 can interact (Figure 6H lane 2,I). These results suggested that MdTPL4 may serve as a linker between MdZFP3 and MdHDA19, allowing MdZFP3, MdTPL4, and MdHDA19 to form a trimeric complex. GUS reporter gene assays in *N. benthamiana* leaves showed that simultaneous transient infection with *35S:MdZFP3*, *35S:MdTPL4*, and *35S:MdHDA19* enhanced the inhibitory effect of MdZFP3 on the *ProMdPG1*, *ProMdPL5*, *ProMdβ‐Gal9*, *ProMdα‐AFase2*, *ProMdXET1*, and *ProMdEXP8* promoters. However, when this effect did not take place in the absence of *35S:MdTPL4* (Figure S6, Supporting Information).

**Figure 7:**
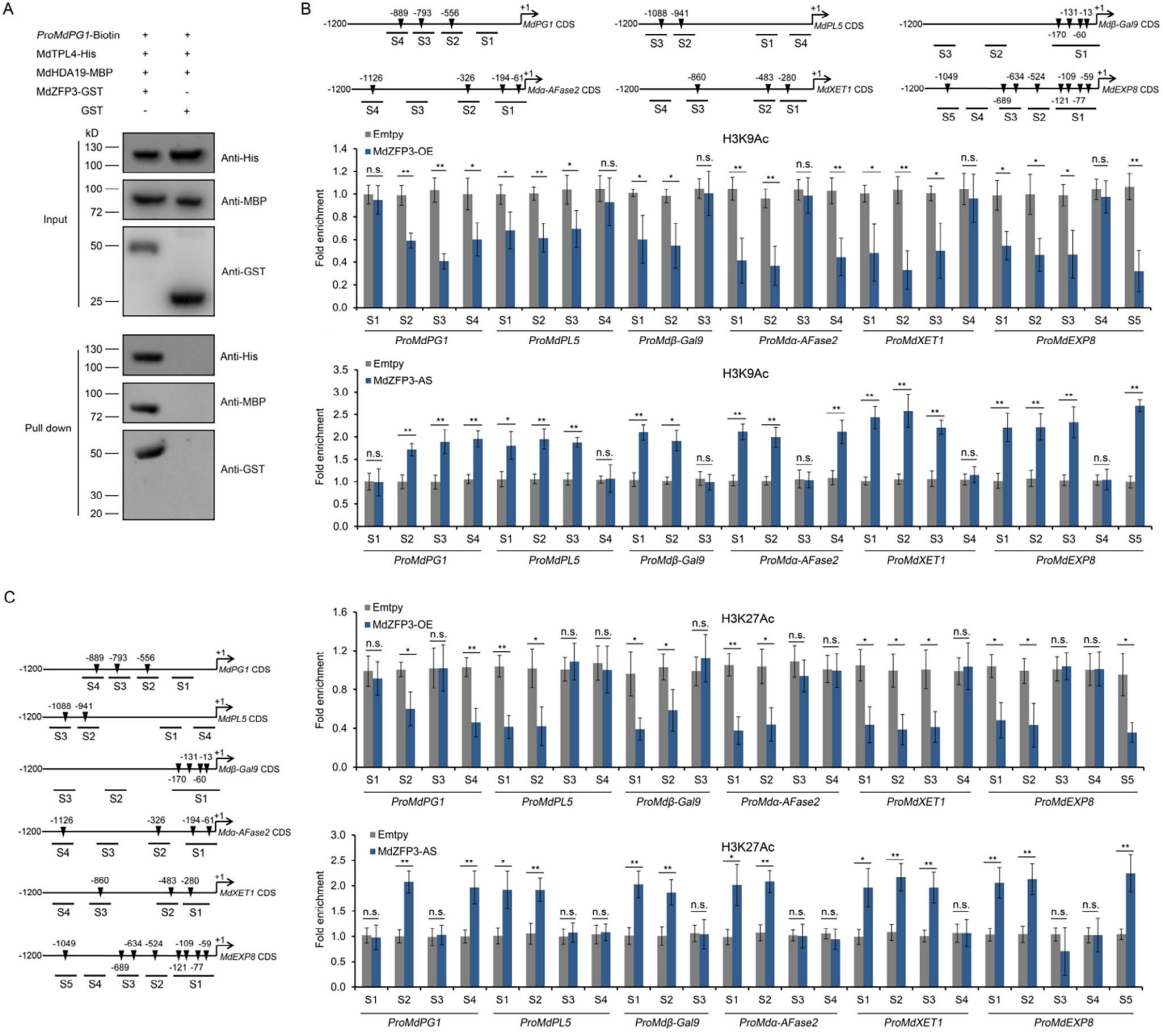
MdZFP3 recruits MdTPL4 and MdHDA19 to the promoter of its downstream genes, downregulating histone acetylation levels. A) DNA pull‐down assay showing that the *MdPG1* promoter is bound by MdTPL4 and MdHDA19 through MdZFP3. In this assay, recombinant MdTPL4‐HIS and MdHDA19‐MBP were incubated with a biotin‐labeled 1000 bp DNA fragment of the *MdPG1* promoter, along with MdZFP3‐GST or GST. The complexes were then pulled down using streptavidin agarose beads. Immunoblots were subsequently probed with anti‐His, anti‐MBP or anti‐GST antibodies. Chromatin immunoprecipitation (ChIP)‐qPCR analysis of B) H3K9Ac and C) H3K27Ac levels in the *MdPG1*, *MdPL5*, *Mdβ‐Gal9*, *Mdα‐AFase2*, *MdXET1*, and *MdEXP8* promoters in *MdZFP3*‐OE and *MdZFP3*‐AS fruit 15 d after harvest. The empty vector transgenic fruits (Empty vector) were used as a control. The data are presented as means ± SE (*n* = 3 groups, 10 transgenic fruit per group). Statistical significance was assessed using Student's *t*‐test (**p < 0.01, *p < 0.05).

Previous studies have reported that transcription factors containing the EAR motif recruit TPL and HDA to the promoters of their downstream target genes.^[^ ^14^, ^25^ ^]^ To determine whether MdZFP3 recruits TPL4 and MdHDA19 to the promoter of its downstream target gene, we used DNA pull‐down assays with a biotin‐labeled *MdPG1* promoter and found that the *MdPG1* promoter only immunoprecipitated MdTPL4 and MdHDA19 in the presence of MdZFP3 (**Figure** **7**A), indicating that MdZFP3 is necessary in recruiting MdTPL4 and MdHDA19 binding to the promoter region of the downstream target genes of MdZFP3. This in turn suggests that MdZFP3 may alter the histone acetylation levels of its target gene promoter by enriching MdTPL4‐MdHDA19 at its target gene promoter, indicative of a transcriptional repression function of MdZFP3. We examined the histone acetylation levels of the promoters of MdZFP3 target genes *MdPG1*, *MdPL5*, *Mdβ‐Gal9*, *Mdα‐AFase2*, *MdXET1*, and *MdEXP8* in fruit with MdZFP3 overexpression (*MdZFP3*‐OE) and silencing (*MdZFP3*‐AS) through ChIP‐qPCR assays, using antibodies that recognize H3K9Ac or H3K27Ac. This revealed that H3K9Ac and H3K27Ac levels in the promoters of the cell wall degradation‐related genes targeted by MdZFP3 were significantly reduced in *MdZFP3*‐OE fruit, while levels were significantly increased in *MdZFP3*‐AS fruit (Figure 7B,C). This indicates the presence of an MdZFP3‐MdTPL4‐MdHDA19 transcriptional repression complex that downregulates the histone acetylation levels of the promoters of cell wall degradation‐related genes targeted by MdZFP3, thereby inhibiting their transcription.

Subsequently, we explored the roles of *MdTPL4* and *MdHDA19* in fruit softening during storage by transiently overexpressing and silencing them in GD fruit, with fruit infiltrated with the empty vector serving as the control (Figure S7A,K and Figure S8A,K, Supporting Information). Our results showed that the expression of *MdTPL4* during storage was significantly increased in *MdTPL4*‐overexpressing (*MdTPL4*‐OE) fruit compared to the control fruit (Figure S7B, Supporting Information), leading to a slower softening rate in *MdTPL4*‐OE fruit (Figure S7C, Supporting Information). Furthermore, the expression levels of genes related to fruit cell wall degradation (*MdPG1*, *MdPL5*, *Mdβ‐Gal9*, *Mdα‐AFase2*, *MdXET1*, and *MdEXP8*) and WSP content were decreased in *MdTPL4*‐OE fruit (Figure S7D–J, Supporting Information). Conversely, transient silencing of *MdTPL4* (*MdTPL4*‐AS fruit) resulted in the opposite effect (Figure S7L–T, Supporting Information). Similar results were observed in fruits overexpressing and silencing *MdHDA19* (Figure S8A–T, Supporting Information). This suggested that *MdTPL4* and *MdHDA19* act as repressors in apple fruit softening.

### 2.7 Ethylene Promotes the Ubiquitination of MdZFP3 by MdEAEL1 during Apple Fruit Storage

**Figure 8:**
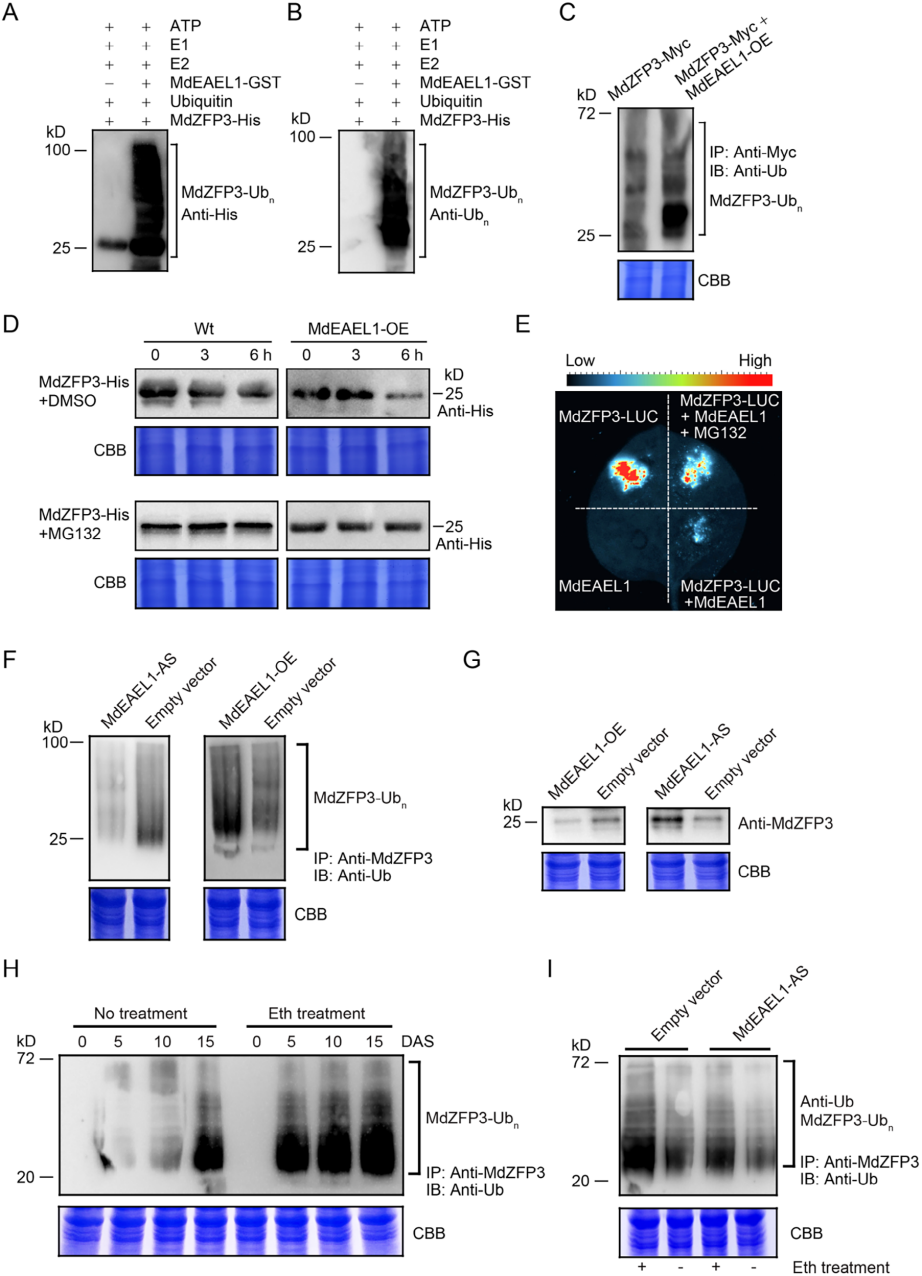
Ethylene promotes the ubiquitination of MdZFP3 by MdEAEL1, leading to MdZFP3 degradation. MdEAEL1 ubiquitination of MdZFP3 in vitro. The presence of ATP, ubiquitin, E1, E2, and recombinant MdEAEL1‐GST allowed the detection of potential E3 ubiquitin ligase activity using MdZFP3‐His as a substrate. Immunoblot analysis was performed to identify the ubiquitination of MdZFP3 using A) anti‐His or B) anti‐ubiquitin (Ub) antibodies. C) MdZFP3 ubiquitination in MdZFP3‐Myc and *MdEAEL1*‐OE + MdZFP3‐Myc apple calli (pretreated with 50 µm MG132). MdZFP3 was immunoprecipitated using an anti‐Myc antibody, and ubiquitinated MdZFP3‐Myc was detected using an anti‐Ub antibody. D) Cell‐free degradation assay performed using protein extracts from transgenic apple calli (*MdEAEL1*‐OE) and wild‐type (Wt) to assess the abundance of recombinant MdZFP3‐His. Immunoblot analysis was performed using an anti‐His antibody to determine MdZFP3‐His levels. MG132, wild‐type (Wt) and *MdEAEL1*‐OE transgenic apple calli were treated separately with 50 µm MG132. E) LUC reporter gene assay indicated that MdEAEL1 mediates the degradation of MdZFP3 through the 26S proteasome pathway. The reporter *35S:MdZFP3:LUC*, alone or together with 35S:MdEAEL1, was infiltrated into *N. benthamiana* leaves to assess LUC activity. MG132, *N. benthamiana* leaves treated with 50 µm MG132. F) Ubiquitination assays of apple fruits silencing *MdEAEL1* (*MdEAEL1*‐AS) or overexpressing *MdEAEL1* (*MdEAEL1*‐OE) after 15 d of storage shows that MdEAEL1 can ubiquitinate MdZFP3. Proteins were extracted from apple pretreated with 50 µm MG132. MdZFP3 protein was immunoprecipitated using an anti‐MdZFP3 antibody. Immunoblotting was performed to detect the ubiquitination of MdZFP3 using an anti‐Ub. G) Abundance of the MdZFP3 protein in *MdEAEL1*‐OE or *MdEAEL1*‐AS apple fruit after 15 d of storage. H) The ubiquitination level of MdZFP3 gradually increased during the apple fruit storage and was enhanced by ethylene treatment. DAS, days after storage. I) The ethylene‐promoted ubiquitination of MdZFP3 was inhibited by silencing of *MdEAEL1*. “+” Ethylene treatment, “−” Non‐treatment. Ubiquitination assay was performed as described in F. In C, D, F, G, H, and I, Coomassie brilliant blue (CBB) staining of total protein extracts served as a control for equal sample loading.

Given that the E3 ubiquitin ligase MdEAEL1 interacts with MdZFP3 (Figure 3A–C), we investigated whether MdEAEL1 ubiquitinates MdZFP3. We performed an in vitro ubiquitination assay and immunoblot analysis using anti‐ubiquitin or anti‐His antibodies to detect multi‐ubiquitinated MdZFP3‐His in the presence of ubiquitin, E1, E2, and E3 (MdEAEL1‐GST). We observed that the ubiquitinated bands disappeared when the reaction mixture lacked MdEAEL1‐GST substrate (**Figure** **8**A,B). Furthermore, in vivo ubiquitination assays were performed using single transgenic Myc‐MdZFP3 overexpressing calli (*35S:Myc‐MdZFP3*) and double transgenic calli overexpressing both Myc‐MdZFP3 and *MdEAEL1*‐OE (*35S:Myc‐MdZFP3*/*35S:MdEAEL1*). Immunoblot analysis revealed that *MdEAEL1*‐overexpression promoted the ubiquitination of MdZFP3 (Figure 8C). Next, we performed in vitro protein degradation assays to determine the effect of MdEAEL1 on the stability of MdZFP3. Protein extracts from transgenic apple calli transformed with *35S:MdEAEL1* (*MdEAEL1*‐OE) or wild‐type (Wt) calli were incubated with recombinant MdZFP3‐His. We observed that the degradation of MdZFP3 was promoted by *MdEAEL1* overexpression and that treatment with MG132 substantially inhibited this degradation (Figure 8D). In addition, we saw a strong luminescence signal in *N. benthamiana* leaves transformed with *35S:MdZFP3‐LUC*. However, the luminescence signal was substantially reduced in leaves overexpressing both *35S:MdZFP3‐LUC* and *35S:MdEAEL1*, and this reduction was inhibited by MG132 treatment (Figure 8E). Collectively, these results suggested that the degradation of MdZFP3 by the 26S proteasome is mediated by the E3 ubiquitin ligase MdEAEL1.

Since ethylene activates *MdEAEL1* transcription during apple fruit storage (Figure 1K), and MdEAEL1 can ubiquitinate and degrade MdZFP3, we investigated whether ethylene affects the ubiquitination and degradation of MdZFP3 by MdEAEL1 during fruit storage. First, we examined the ubiquitination of MdZFP3 in *MdEAEL1*‐OE and *MdEAEL1*‐AS fruit after 15 d of storage. Ubiquitination levels of MdZFP3 were higher in *MdEAEL1*‐OE fruit and notably decreased in the *MdEAEL1*‐AS fruit (Figure 8F). We also assessed the protein abundance of MdZFP3 in *MdEAEL1*‐OE fruit and *MdEAEL1*‐AS fruit and found that levels of MdZFP3 were notably lower in *MdEAEL1*‐OE fruit, and higher in *MdEAEL1*‐AS fruit (Figure 8G). These results suggested that MdEAEL1 ubiquitinates and degrades MdZFP3 during fruit storage, and we also found that ethylene treatment significantly promoted the level of MdZFP3 ubiquitination during storage (Figure 8H). However, when *MdEAEL1* was silenced, the ethylene‐induced ubiquitination of MdZFP3 was suppressed (Figure 8I), indicating that the ability of ethylene to promote MdZFP3 ubiquitination is at least partially dependent on MdEAEL1. Together, these results suggest that ethylene‐induced MdEAEL1 promotes the ubiquitination and degradation of MdZFP3 during fruit storage.

### 2.8 MdEAEL1 Destabilized MdZFP3, Leading to Disassembly of the MdZFP3‐MdTPL4‐MdHDA19 Complex and Elevated Acetylation Levels in the Promoters of Cell Wall Degradation‐Related Genes

**Figure 9:**
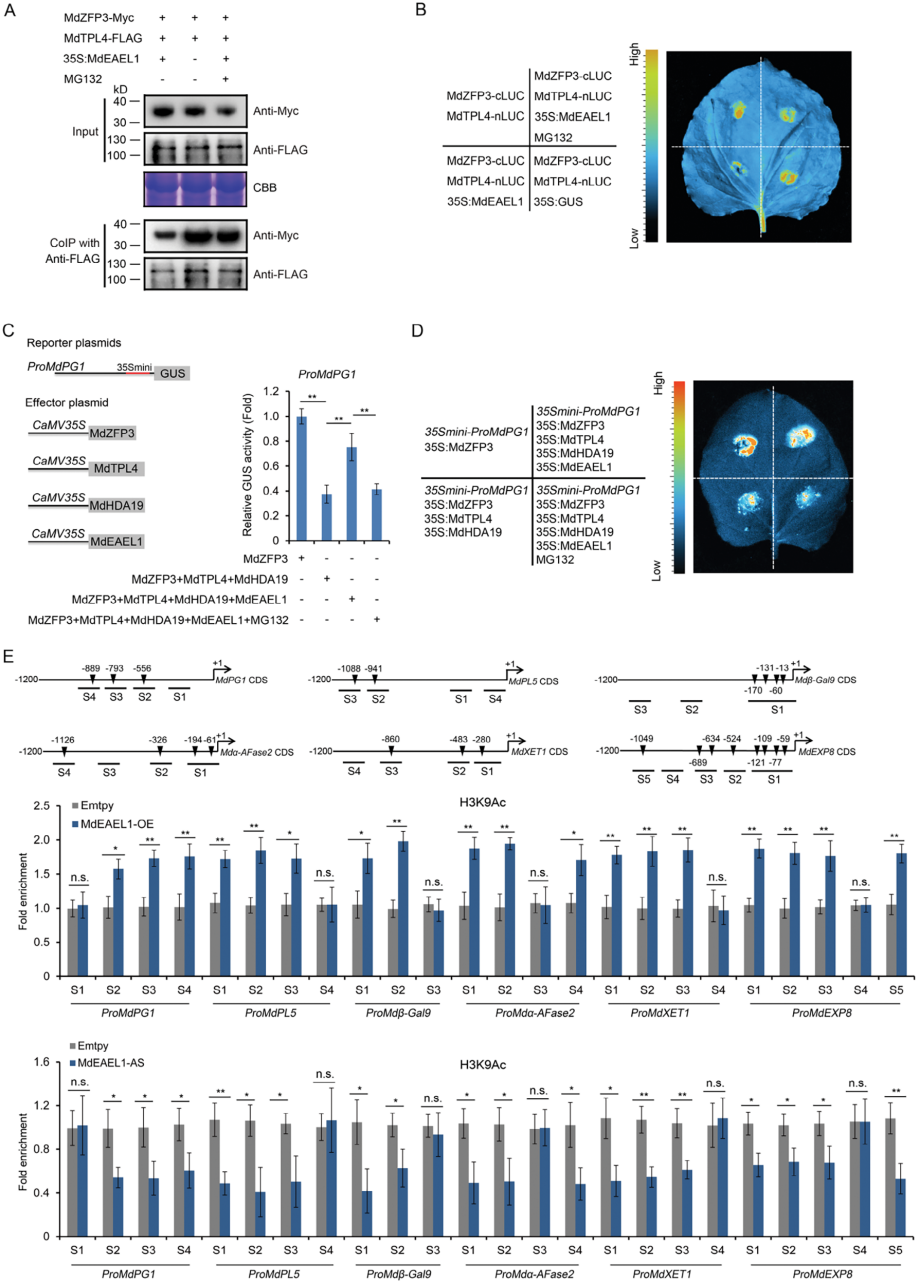
MdEAEL1 mediates the disassembly of the MdZFP3‐MdTPL4‐MdHDA19 transcriptional repression complex, upregulating histone acetylation levels in the promoter region of MdZFP3 target genes. A) MdEAEL1 inhibits the interaction between MdZFP3 and MdTPL4 in a co‐IP assay. MdZFP3 tagged with Myc (MdZFP3‐Myc) and FLAG‐fused MdTPL4 (MdTPL4‐FLAG) were co‐expressed together with *35S:EAEL1* expressed in *N. benthamiana* leaves. As a control, only MdZFP3‐Myc and MdTPL4‐FLAG were co‐expressed. The immunoprecipitation analysis was conducted using an anti‐FLAG antibody and immunoblotting was performed using anti‐FLAG and anti‐Myc antibodies. Coomassie brilliant blue (CBB) staining of total protein extracts served as a control for equal sample loading. B) MdTPL4 inhibition of the interaction between MdZFP3 and MdTPL4 in *N. benthamiana* leaves assessed by luciferase complementation imaging (LCI). C) GUS reporter assays indicating that MdEAEL1 mediates the disassembly of the MdZFP3‐MdTPL4‐MdHDA19 complex, promoting the transcription of *MdPG1*. The GUS reporter plasmid was co‐transfected into *N. benthamiana* leaf together with individual effector plasmids. D) The LUC reporter was co‐transfected into *N. benthamiana* leaves together with individual effector plasmids. E) Chromatin immunoprecipitation (ChIP)‐qPCR analysis of H3K9Ac level at the *MdPG1*, *MdPL5*, *Mdβ‐Gal9*, *Mdα‐AFase2*, *MdXET1*, and *MdEXP8* promoters in *MdEAEL1*‐OE and *MdEAEL1*‐AS fruit at 15 d after harvest. The empty vector transgenic fruit (Empty vector) were used as a control. The data statistical analysis was used as described in Figure 7B. In A–D, MG132 was used as a proteasome inhibitor.

Given that MdEAEL1 mediates the ubiquitination and degradation of MdZFP3, we hypothesized that this process may lead to the disassembly of the MdZFP3‐MdTPL4‐MdHDA19 transcriptional repression complex. A co‐IP assay with transient infection of *N. benthamiana* leaves (*35S:Myc‐MdZFP3*/*35S:FLAG‐MdTPL4*/*35S:MdEAEL1* or *35S:Myc‐MdZFP3*/*35S:FLAG‐MdTPL4*) demonstrated that MdEAEL1 inhibited the interaction between MdZFP3 and MdTPL4, and this inhibitory effect could be reversed by treatment with MG132 (**Figure** **9**A), indicating that MdEAEL1 mediates the ubiquitination and degradation of MdZFP3, weakening the interaction between MdZFP3 and MdTPL4. This was further supported using an LCI reporter experiment (Figure 9B). Together, these results indicate that MdEAEL1 mediates the ubiquitination and degradation of MdZFP3, leading to the disassembly of the MdZFP3‐MdTPL4‐MdHDA19 transcriptional repression complex. We hypothesized that this process may affect the transcriptional repression of *MdPG1*, *MdPL5*, *Mdβ‐Gal9*, *Mdα‐AFase2*, *MdXET1*, and *MdEXP8* by the MdZFP3‐MdTPL4‐MdHDA19. Indeed, we found that the co‐expression of *MdZFP3‐MdTPL4‐MdHDA19* with the respective promoters of these genes in a GUS reporter gene assay, led to a substantial reduction in GUS activity, and that this effect was reversed by co‐expression with *MdEAEL1*. Furthermore, the application of MG132 resulted in a persistent MdZFP3‐MdTPL4‐MdHDA19‐dependent suppression of *MdPG1*, *MdPL5*, *Mdβ‐Gal9*, *Mdα‐AFase2*, *MdXET1*, and *MdEXP8* expression (Figure 9C and Figure S9A, Supporting Information). A LUC reporter gene experiment further confirmed this result (Figure 9D and Figure S9B, Supporting Information). These data indicate that MdEAEL1 mediates the ubiquitination and degradation of MdZFP3, leading to the disassembly of the MdZFP3‐MdTPL4‐MdHDA19 complex and consequent reduced repression of genes related to fruit cell wall degradation. In addition, we conducted ChIP‐qPCR assays to assess the histone acetylation levels of the promoters of MdZFP3‐MdTPL4‐MdHDA19 target genes (*MdPG1*, *MdPL5*, *Mdβ‐Gal9*, *Mdα‐AFase2*, *MdXET1*, and *MdEXP8*) in both *MdEAEL1*‐OE and *MdEAEL1*‐AS fruit, using antibodies specific for H3K9Ac and H3K27Ac. We found that the levels of H3K9Ac and H3K27Ac were significantly higher in *MdEAEL1*‐OE fruit and markedly lower in *MdEAEL1*‐AS fruit (Figure 9E and Figure S10, Supporting Information). This indicates that MdEAEL1 can alter the histone acetylation levels of the promoters of fruit cell wall degradation‐related genes targeted by the MdZFP3‐MdTPL4‐MdHDA19 transcriptional repression complex.

### 2.9 The MdEAEL1‐MdZFP3‐MdTPL4‐MdHDA19 Module Forms a Feedback Loop that Suppresses the Transcriptional Repression Activity of MdZFP3 on the *MdEAEL1* Promoter

**Figure 10:**
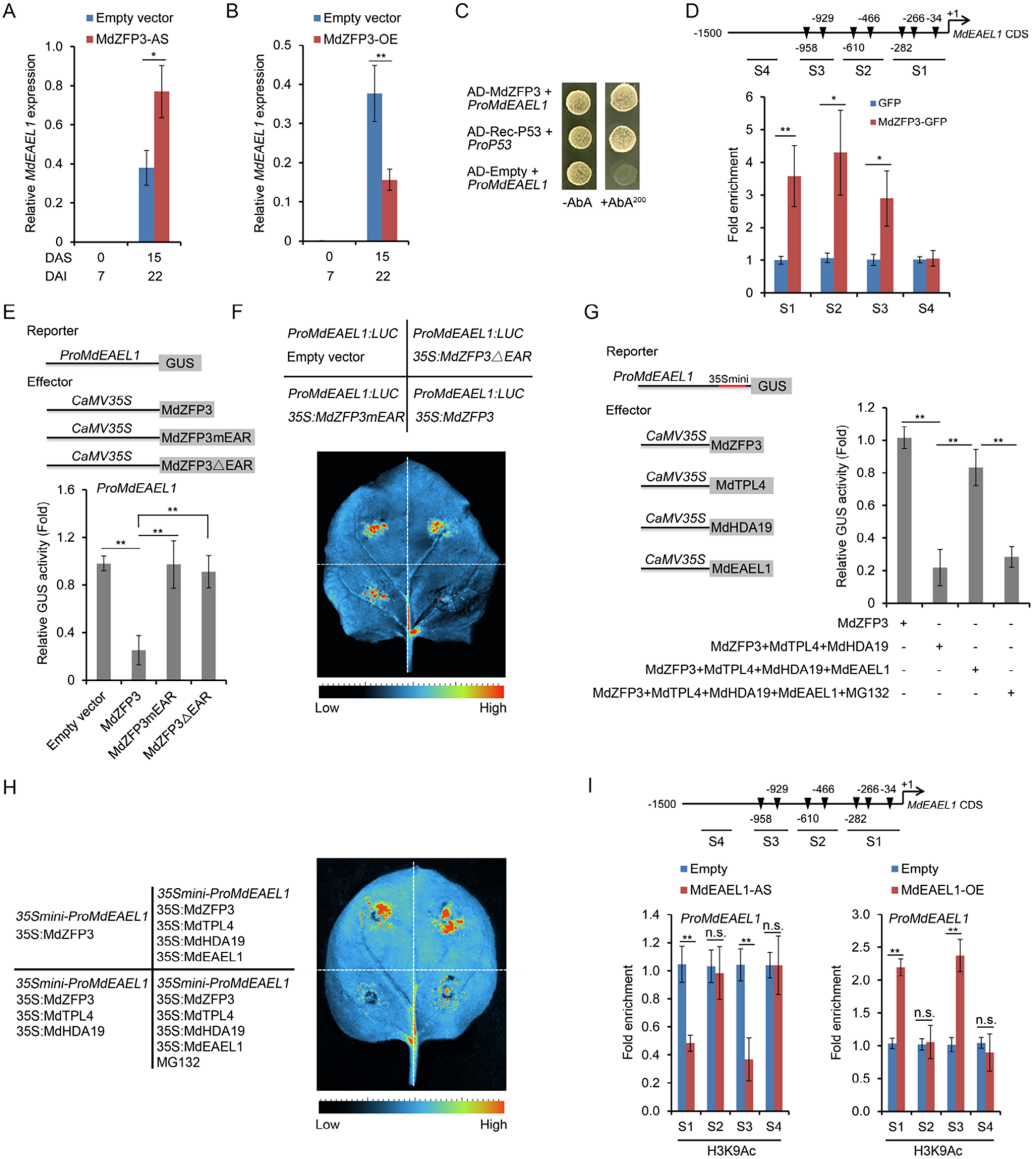
MdEAEL1‐MdZFP3‐MdTPL4‐MdHDA19 module forms a feedback loop that suppressed the transcriptional repression activity of MdZFP3 on the *MdEAEL1* promoter. Reverse transcription‐quantitative PCR (RT‐qPCR) analysis of the expression of *MdEAEL1* in the fruit of A) *MdZFP3*‐AS and B) *MdZFP3*‐OE. The data statistical analysis was used as described in Figure 2. C) Yeast one‐hybrid (Y1H) assay showing that MdZFP3 directly binds to *MdEAEL1* promoter. The basal concentration of AbA (aureobasidin A) used was 200 ng mL^−1^. D) Chromatin immunoprecipitation (ChIP)‐qPCR assay demonstrating the in vivo binding of MdZFP3 to the *MdEAEL1* promoter. The data statistical analysis was used as described in Figure 5B. E) GUS reporter assays showing that MdZFP3 depends on an EAR motif to inhibit the expression of *MdEAEL1*. The GUS reporter plasmid was co‐transfected into *N. benthamiana* leavestogether with individual effector plasmids. F) The LUC reporter was co‐transfected into *N. benthamiana* leaves together with individual effector plasmids. In E and F, MdZFP3mEAR, the amino acids LGLDL in the EAR motif of MdZFP3 were mutated to SDSDS. MdZFP3△EAR, MdZFP3 with a deleted EAR motif. G) GUS reporter assays indicating that MdEAEL1 mediates the disassembly of the MdZFP3‐MdTPL4‐MdHDA19 complex, promoting the transcription of *MdEAEL1*. The GUS reporter plasmid was co‐transfected into *N. benthamiana* leaves together with individual effector plasmids. MG132 was used as a proteasome inhibitor. In E and G, the data statistical analysis was used as described in Figure 5C. H) The LUC reporter was co‐transfected into *N. benthamiana* leaves together with individual effector plasmids. MG132 was used as a proteasome inhibitor. I) ChIP analysis of H3K9Ac level at the *MdEAEL1* promoter in *MdEAEL1*‐OE and *MdEAEL1*‐AS fruit at 15 d after harvest. The empty vector transgenic fruit (Empty vector) were used as a control. The data statistical analysis was used as described in Figure 7B.

We the evaluated the expression of *MdEAEL1* in apple fruit with silenced or elevated *MdZFP3* expression during storage and found that its expression was higher in the silenced fruits than in the control fruit, and lower in *MdZFP3* overexpressing fruits (**Figure** **10**A,B), indicating that MdZFP3 may regulate the expression of *MdEAEL1*. We detected the presence of the MdZFP3 binding element A[AG/CT]CNAC in the promoter region of *MdEAEL1* (Figure S4, Supporting Information), and hypothesized that MdZFP3 directly binds to the promoter of *MdEAEL1*, thereby regulating its transcription. A Y1H assay showed that MdZFP3 directly binds to the *MdEAEL1* promoter (Figure 10C), and this was confirmed through in vivo experiments using a ChIP‐qPCR assay in transgenic apple calli, which showed that MdZFP3 directly bound to the S1/S2/S3 region of the *MdEAEL1* promoter (Figure 10D). A GUS reporter gene assay in *N. benthamiana* leaves showed that co‐transformation of *Pro35S:MdZFP3* with the *MdEAEL1* promoter fused to the GUS gene (*ProMdEAEL1:GUS*) resulted in a significant reduction in the GUS signal. In contrast, co‐transformation *Pro35S:MdZFP3mEAR* or *Pro35S:MdZFP3△EAR* with *ProMdEAEL1:GUS* did not result in a significant change in GUS accumulation (Figure 10E). This indicated that the inhibition of the transcriptional activity of the *MdEAEL1* promoter by MdZFP3 is dependent on the EAR motif, and this was further supported by a LUC reporter gene experiment further (Figure 10F). Interestingly, we observed that the simultaneous expression of *MdZFP3‐MdTPL4‐MdHDA19* alongside the *MdEAEL1* promoter in a GUS reporter gene assay caused a significant decrease in GUS activity. This decrease was counteracted when *MdEAEL1* was co‐expressed. Moreover, treatment with MG132 led to a sustained downregulation of the activity of *MdEAEL1* promoter (Figure 10G). A LUC reporter gene experiment further confirmed this result (Figure 10H). These data indicated that MdEAEL1 mediates the ubiquitination and degradation of MdZFP3, leading to the disassembly of the MdZFP3‐MdTPL4‐MdHDA19 complex, inhibiting its transcriptional repression of *MdEAEL1*.

Next, we performed ChIP‐qPCR assays to evaluate the histone acetylation levels of the *MdEAEL1* promoter in both *MdEAEL1*‐OE and *MdEAEL1*‐AS fruit, utilizing antibodies specific for H3K9Ac or H3K27Ac. We observed that H3K9Ac and H3K27Ac levels at the *MdEAEL1* promoter were significantly reduced in *MdEAEL1*‐AS fruit, and elevated in *MdEAEL1*‐OE fruit (Figure 10I and Figure S11, Supporting Information), indicating that MdEAEL1 alters the histone acetylation level of its own promoter. Taken together, these results indicate that MdEAEL1 mediates the ubiquitination and degradation of the MdZFP3‐MdTPL4‐MdHDA19 transcriptional repression complex, which can upregulate the histone acetylation levels at the *MdEAEL1* promoter, forming a feedback loop that promotes its own transcription.

## 3 Discussion

Ethylene is a key factor causing postharvest senescence and softening of climacteric fruits. It can induce the expression of genes related to senescence and softening in fruit, such as *MdACS1*, *MdACO1*, and *MdPG1*, thereby promoting fruit senescence and softening, leading to a shortened shelf life of fruits, and exacerbating postharvest losses.^[^ ^8^, ^26^ ^]^ Therefore, a detailed analysis of the ethylene signal transduction pathway in postharvest fruits is crucial. Here, we identified an E3 ubiquitin ligase gene, *MdEAEL1*, that similarly showed specific postharvest expression. We determined that ethylene‐induced MdEAEL1 regulates the transcription of genes related to cell wall degradation by modulating the stability of the MdZFP3‐MdTPL4‐MdHDA19 complex, thereby affecting fruit softening. In this pathway, MdEAEL1 acts as a key element in ethylene signal transduction and represents a novel ethylene signal transducer.

Studies have shown that C2H2‐type ZFPs are involved in the postharvest ripening of fruits. For example, in tomato, SlZFP2 directly regulates the transcription of ABA biosynthesis‐related and fruit‐ripening‐related regulatory factor genes, thereby inhibiting fruit ripening.^[^ ^27^ ^]^ In banana, a MaC2H2‐Like transcription factor has been shown to directly target the promoters of cell wall degradation‐related genes, thereby promoting their transcription and increasing fruit softening.^[^ ^21^ ^]^ This suggests that the transcriptional regulatory role of C2H2 transcription factors in regulating fruit ripening and softening has been confirmed. However, the mechanism by which C2H2 regulates the activity of target gene promoters has yet to be elucidated. Here, we also found that the C2H2‐type transcription factor MdZFP3 directly regulates the transcription of cell wall degradation‐related genes (*MdPG1*, *MdPL5*, *Mdβ‐Gal9*, *Mdα‐AFase2*, *MdXET1*, and *MdEXP8*), inhibiting their transcription. The genes related to cell wall degradation that are targeted by MdZFP3 are induced by ethylene during fruit storage. Specifically, the transcription of *MdPL5* was not promoted by ethylene treatment on days 5 and 10 of storage, but it was inhibited by 1‐MCP treatment. This suggests that a certain threshold of ethylene is required to promote the transcription of *MdPL5*. Among these cell wall degradation‐related genes, the genetic evidence showing that *MdPG1* promotes apple fruit softening by changing cell wall composition and structure has been well elucidated.^[^ ^8^, ^9^ ^]^ However, further analysis is still needed to understand the genetic evidence of other cell wall degradation‐related genes in regulating apple fruit softening. This study found that MdZFP3 can directly bind to *MdPG1* promoter and regulate its transcription, which sufficiently demonstrates the crucial role of MdZPF3 as a key transcription factor in regulating apple fruit softening. Interestingly, MdZFP3 contains an EAR motif at the C‐terminal end, giving MdZFP3 a negative regulatory function. MdZFP3 forms a transcriptional repression complex with TPL4‐MdHDA19, altering the levels of histone acetylation of MdZFP3 target gene promoters, thereby regulating gene transcription. We have elucidated the epigenetic mechanism mediated by MdZFP3 involved in gene regulation, which in turn regulates fruit softening. A similar mechanism also occurs in the SlERF.F12 tomato fruit senescence process, where SlERF.F12 interacts with SlTPL2 through its EAR domain to form a transcriptional repression complex with SlTPL2‐SlHDA1/3, altering the acetylation levels of histones at the promoter regions of cell wall degradation‐related genes, thereby regulating gene transcription.^[^ ^14^ ^]^ This suggests that the EAR motif‐mediated epigenetic regulation ethylene‐induced softening may be conserved among climacteric fruit. However, unlike the SlERF.F12‐SlTPL2‐SlHDA1/3 transcriptional repression complex found in tomato, MdTPL4 acts as an intermediary that connects MdZFP3 and MdHDA19, forming a complex rather than interacting with each other. This suggests variations in the formation of transcriptional repression complexes mediated by the EAR motif among different types of fruits.

It was previously reported that the transcriptional repression complex mediated by the EAR motif regulates the transcription of downstream genes by changing its stability through ubiquitination modification, serving as a key mechanism by which plants respond to plant hormones.^[^ ^15^ ^]^ For example, NINJA (JAZ^EAR^‐TPL) complex relies on jasmonic acid signaling for its ubiquitination and degradation.^[^ ^28^ ^]^ In addition, the AUX/IAA^EAR^‐TPL complex is known to be degraded via the proteasomal degradation pathway in an auxin dependent manner.^[^ ^29^ ^]^ However, no reports have yet linked this mechanism to ethylene signal transduction. In this study, we discovered that the E3 ubiquitin ligase MdEAEL1 can ubiquitinate and degrade the transcription factor MdZFP3 containing the EAR motif, leading to the degradation of the transcriptional repression complex MdZFP3^EAR^‐MdTPL4‐MdHDA19, transmitting the ethylene signal, which is responsible for fruit softening. This illustrates how post‐translational modification and epigenetic modification of a transcription factor can collaboratively regulate postharvest fruit softening. Unfortunately, due to the long juvenile period of apple trees, it is challenging to obtain direct transgenic fruit. This study only used transient gene expression method to validate the gene functions of *MdEAEL1*, *MdZFP3*, *MdTPL4*, and *MdHDA19* in regulating apple fruit softening. Therefore, further exploration is needed to provide direct genetic evidence.

It was previously reported that the C2H2‐type ZFP transcription factor, OsZFP252, regulates the transcription of the E3 ubiquitin ligase gene, *OsRING1*, and is involved in drought stress responses in rice (*Oryza sativa*).^[^ ^30^ ^]^ Here, we found that MdZFP3 directly inhibits the transcription of *MdEAEL1* by recruiting MdTPL4‐MdHDA19 to form a trimeric transcriptional repression complex, which alters the histone acetylation levels in the *MdEAEL1* promoter. Ethylene‐induced MdEAEL1 can mediate the ubiquitination and degradation of MdZFP3, leading to the disassembly of the MdZFP3‐MdTPL4‐MdHDA19 transcriptional repression complex, thereby promoting the transcription of *MdEAEL1* itself. This indicates that the MdEAEL1 and MdZFP3‐MdTPL4‐MdHDA19 module participates in a feedback loop to regulate the transcription of *MdEAEL1*, thereby amplifying the promoting effect of MdEAEL1 on the expression of cell‐wall degradation‐related genes. Such a mode of action would presumably allow rapid fruit responses to ethylene and postharvest softening. In addition, our previous research found that the E3 ubiquitin ligase MdPUB24 mediates the degradation of the transcriptional repressor MdBEL7, which contains an EAR motif, and plays a role in the ethylene‐induced de‐greening of postharvest apple fruit.^[^ ^31^ ^]^ Here, we found that MdZFP3 is ubiquitinated and degraded by the E3 ubiquitin ligase MdEAEL1, thereby promoting post‐harvest softening. This indicates that the ubiquitination and degradation of EAR‐type transcriptional repressors mediated by E3 ubiquitin ligase are involved in multiple aspects of post‐harvest fruit de‐greening and softening, and constitute an important regulatory mechanism for post‐harvest fruit senescence.

**Figure 11:**
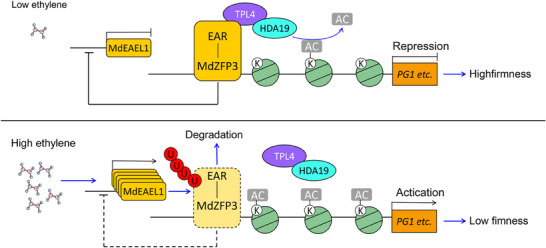
A model showing the ethylene‐activated E3 ubiquitin ligase MdEAEL1 mediating the disassembly of the MdZFP3‐MdTPL4‐MdHDA19 transcriptional repression complex, thereby forming a loop that promotes apple fruit softening. During storage, ripe apples with low ethylene levels maintain the stability of the MdZFP3‐TPL4‐HDA19 transcriptional repression complex, which prevents the transcription of cell wall degradation‐related genes. As ethylene production increases after storage, ethylene‐activated MdEAEL1 mediates the ubiquitination and degradation of MdZFP3, leading to the disassembly of the MdZFP3‐MdTPL4‐MdHDA19 transcriptional repression complex. This upregulates the acetylation levels of histones in the promoter regions of cell wall degradation‐related genes, resulting in elevated transcription levels of these genes and leading to fruit softening. The disassembly of the MdZFP3‐MdTPL4‐MdHDA19 complex triggers the transcription of *MdEAEL1*, forming a feedback loop that further promotes fruit softening.

In summary, we found that the C2H2‐type transcription factor MdZFP3 relies on the EAR motif to form a transcriptional repression complex with MdTPL4‐HDA19, which downregulates the histone acetylation levels in the promoter regions of cell wall degradation‐related genes, thereby inhibiting their transcription. Furthermore, we determined that ethylene‐activated MdEAEL1 mediates the ubiquitination and degradation of MdZFP3, leading to the disassembly of the MdZFP3‐MdTPL4‐MdHDA19 repression complex, thereby increasing the transcription of cell wall degradation‐related genes and facilitating softening. In addition, the disassembly of the MdZFP3‐MdTPL4‐MdHDA19 complex promotes the transcription of *MdEAEL1* itself, forming a feedback loop that further enhances fruit softening (**Figure** **11**). Our study integrates the mechanisms of post‐translational regulation and epigenetic regulation of transcription factor in the ethylene‐mediated softening of apple fruit during storage. This discovery offers new insights into fruit ripening, which could lead to the development of strategies for extending the shelf life of fruits.

## 4 Experimental Section

### Plant Materials and Treatments

Apple (*Malus domestica* cv. Golden Delicious; GD) fruit were harvested from the Liaoning Pomology Institute orchard (Xiongyue, China) at 20, 50, 80, 110, and 140 d after full bloom. In this region, the fruit reach physiological maturity 140 d after full bloom.^[^ ^32^ ^]^ Mature fruit were treated with 1‐MCP and ethylene, and untreated fruit were used as controls. These fruit were stored at room temperature (24 °C) for 20 d, with samples taken every 5 d. For the 1‐MCP treatment, the fruit were placed in a sealed container and exposed to 1 µL L^−1^ of 1‐MCP (Fresh Doctor, China) for 12 h at room temperature. For the ethylene treatment, the fruits were immersed in a 0.1% [v/v] ethephon solution (A600453, Sangon Biotech, China) for 15 s and then placed in a sealed container for 12 h at room temperature. Apple fruit calli and *N. benthamiana* plants were cultivated in the laboratory as previously described.^[^ ^33^ ^]^ For MG132 (26S proteasome inhibitor) treatment, apple calli were immersed in liquid Murashige and Skoog (MS) medium containing 50 µm MG132 and gently shaken at 24 °C for 12 h. The *N. benthamiana* leaves were infiltrated with 50 µm MG132 and kept in darkness for 12 h. Apple fruit were dissected into 1 mm thick, 10 mm diameter sections, which were then submerged in MS medium containing 50 µm MG132 for 12 h.

### Measurement of Fruit Firmness, Ethylene Production, and Water‐Soluble Pectin

To measure the firmness of the apples, a TA.XT Plus Texture Analyzer (Stable Micro Systems, UK) was used according as previously described.^[^ ^7^, ^34^ ^]^ To measure ethylene production, individual fruit were placed in a 1000 mL airtight bottle with a septum at 24 °C for 1 h, then a 1 mL sample of headspace gas was withdrawn using a syringe for analysis using a gas chromatograph (7890A; Agilent Technology, NY, USA) with a flame ionization detector.^[^ ^33^ ^]^ The measurement of water‐soluble pectin content was performed as previously described.^[^ ^34^ ^]^ In brief, extract cell wall materials (CWM) from 3 g of frozen apple fruit flesh and separate water‐soluble pectin (WSP) from 50 mg of CWM. Determine the WSP content using the carbazole‐ethanol method.

### RT‐qPCR

A modified cetyltrimethylammonium bromide (CTAB) method was used for total RNA extraction from apple fruit, as previously described.^[^ ^35^ ^]^ First‐strand cDNA was synthesized from 1 µg of total RNA using PrimeScript RT reagent Kit (Cat. no. RR047, TaKaRa, Japan). qPCR analysis was performed using a 2 × UltraSYBR Mixture (Cat. no. CW2601; CWBIO, China) on a qTOWER3 G PCR System (Analytik Jena, Germany) as previously described.^[^ ^36^ ^]^ Primers used for this study are listed in Table S3 (Supporting Information). *Mdactin* (EB136338) was used as the reference gene for expression normalization.^[^ ^7^ ^]^

### Protein Extraction and Immunoblot Analysis

Protein was extracted from apple calli and fruit, then analyzed using immunoblotting as previously described.^[^ ^36^ ^]^ The protein concentration in each sample was measured using a BCA protein assay kit (Cat. no. P0012S, CWBIO). Coomassie Brilliant Blue staining of total protein extracts served as a control to confirm equal gel loading across samples. Purified recombinant MdZFP3 was used to generate a specific antibody in rabbit (Sangon Biotech, Shanghai, China). Antibodies including anti‐GFP (Cat. no. HT801; TransGen Biotech, Beijing, China), anti‐Myc (Cat. no. HT101; TransGen Biotech), anti‐FLAG (Cat. no. 14793S; Cell Signaling Technology, USA), anti‐His (Cat. no. HT501; TransGen Biotech), anti‐GST (Cat. no. HT601; TransGen Biotech), anti‐MBP (Cat. no. HT701; TransGen Biotech), and anti‐ubiquitin (‐Ub) (Cat. no. ST1200, Sigma, USA) were each diluted 1:1000 with Tris‐buffered saline containing Tween 20 (TBST buffer, 20 mm Tris‐HCl, pH 7.5, 150 mm NaCl, and 0.1% [v/v] Tween 20) and incubated with nitrocellulose membranes (Cat. no. S80209, Pall Corporation, USA). Horseradish peroxidase‐conjugated secondary antibodies (goat anti‐mouse or anti‐rabbit antibodies; Cat. no. HS201 or HS101, TransGen Biotech) were diluted to 1:3000 in TBST buffer.

### Y2H Assay

Total RNA from ripe apple fruit was used to construct a cDNA library using the “Make Your Own “Mate & Plate” Library System User Manual” (Clontech, CA, USA). *MdEAEL1* or *MdZFP3* served as the bait gene for screening, and its coding sequence (CDS) was cloned into the pGBKT7 vector. Screening for the interacting proteins was employed using SD/‐Ade/‐His/‐Leu/‐Trp/X‐α‐gal medium. For the Y2H assay, the CDS of *MdZFP3*, *MdTPL4*, or *MdHDA19* was inserted into the pGADT7 vector and the CDS of *MdEAEL1*, *MdZFP3*, *MdZFP3mEAR*, *MdZFP3△ZFP3*, or *MdHDA19* was cloned into pGBKT7. The Matchmaker Gold Yeast two‐hybrid system (Clontech, USA) protocol was used to detect protein interactions.

### Co‐IP Assay

For the co‐immunoprecipitation (co‐IP) assay, the CDSs of *MdEAEL1* and *MdHDA19* were cloned into a pRI101‐GFP vector, which contains the GFP sequence and the CaMV 35S promoter, resulting in the *35S:GFP‐MdEAEL1* and *35S:GFP‐MdHDA19* plasmids. Similarly, the CDS of *MdZFP3*, *MdZFP3mEAR*, and *MdZFP3△EAR* was inserted into a pRI101‐Myc vector under the 35S promoter to create the *35S:Myc‐MdZFP3*, *35S:Myc‐MdZFP3mEAR*, and *35S:Myc‐MdZFP3△EAR* plasmids. Lastly, the CDS of *MdTPL4* was placed ligated into a pRI101‐FLAG vector, also containing the 35S promoter, resulting in the *35S:FLAG‐MdTPL4* plasmid. Subsequently, the resulting plasmids *35S:GFP‐MdEAEL1* and *35S:Myc‐MdZFP3*, *35S:Myc‐MdZFP3* and *35S:FLAG‐MdTPL4*, *35S:Myc‐MdZFP3mEAR* and *35S:FLAG‐MdTPL4*, *35S:Myc‐MdZFP3△EAR* and *35S:FLAG‐MdTPL4*, or *35S:FLAG‐MdTPL4* and *35S:GFP‐MdHDA19*, were co‐transformed into apple calli according to a previously described protocol.^[^ ^7^ ^]^ Following the transformation, transgenic calli (*35S:GFP‐MdEAEL1* and *35S:Myc‐MdZFP3*) was subjected to a 12 h treatment with 50 µm MG132 to preserve the stability of the MdZFP3 protein. Calli co‐transformed with *35S:Myc‐MdZFP3* and *35S:GFP*, or *35S:FLAG‐MdTPL4* and *35S:GFP* served as negative controls. The plasmids *35S:Myc‐MdZFP3* and *35S:GFP‐MdHDA19* or *35S:Myc‐MdZFP3* and *35S:FLAG‐MdTPL4* were co‐transformed into *N. benthamiana* leaves as previously described.^[^ ^7^ ^]^ To investigate whether *MdZFP3*, *MdTPL4*, and *MdHDA19* form a transcriptional repression complex, the CDS of *MdTPL4* was ligated into the pRI101 vector to construct the *35S:MdTPL4* plasmid. Following transient overexpression of *35S:MdTPL4* in *N. benthamiana* leaves (*35S:Myc‐MdZFP3* and *35S:GFP‐MdHDA19*), using previously published methods,^[^ ^7^ ^]^ the interactions between MdZFP3 and MdHDA19 were assessed by co‐IP. To investigate the effect of MdEAEL1 on the interaction between MdZFP3 and MdTPL4, the CDS of *MdEAEL1* was cloned into pRI101 to construct the *35S:MdEAEL1* plasmid. Similarly, *35S:MdEAEL1* was transiently overexpressed in *N. benthamiana* leaves (*35S:Myc‐MdZFP3* and *35S:FLAG‐MdTPL4*) and the interaction between MdZFP3 and MdTPL4 was examined through co‐IP. For MG132 treatment, the *N. benthamiana* leaves were infiltrated with 50 µm MG132 and kept in darkness for 12 h. The co‐IP analysis was conducted on transgenic calli or *N. benthamiana* leaves according as previously described.^[^ ^7^ ^]^ A Pierce Co‐immunoprecipitation (IP) kit (Cat. no. 26149, Thermo Scientific) was used to immunoprecipitate proteins, followed by analysis of the precipitated proteins by immunoblotting.

### Firefly LCI Assay

The CDSs of *MdEAEL1*, *MdTPL4*, or *MdZFP3* were cloned into the pCAMBIA1300‐nLuc vector^[^ ^37^ ^]^ to generate the plasmids *35S:MdEAEL1‐nLuc, 35S:MdTPL4‐nLuc, 35S:ZFP3‐nLuc*. The CDS of *MdZFP3*, *MdZFP3mEAR*, *MdZFP3△EAR*, or *MdHDA19* were inserted into the pCAMBIA1300‐cLuc vector to produce the plasmids *35S:MdZFP3‐cLuc*, *35S:MdZFP3*mEAR*‐cLuc*, *35S:MdZFP3*△EAR*‐cLuc*, or *35S:MdHDA19‐cLuc*. The plasmids *35S:MdEAEL1‐nLuc* and *35S:MdZFP3‐cLuc*, *35S:MdTPL4‐nLuc* and *35S:MdTPL4‐nLuc*, *35S:MdTPL4‐nLuc* and *35S:MdZFP3‐cLuc*, *35S:MdTPL4‐nLuc* and *35S:MdZFP3*mEAR*‐cLuc*, *35S:MdTPL4‐nLuc* and *35S:MdZFP3*△EAR‐*cLuc*, or *35S:ZFP3‐nLuc* and *35S:MdHDA19‐cLuc* were then infiltrated into *N. benthamiana* leaves respectively, as previously described.^[^ ^38^ ^]^ To investigate the interaction between MdEAEL1 and MdZFP3, *N. benthamiana* leaves were treated with 50 µm of MG132 12 h prior to LUC imaging. Luciferase activity was assessed as previously described.^[^ ^7^ ^]^ To investigate whether MdZFP3, MdTPL4, and MdHDA19 form a transcriptional repression complex, the CDS of *MdTPL4* was ligated into the pRI101 vector to construct the *35S:MdTPL4* plasmid. Following transient overexpression of *35S:MdTPL4* in *Nicotiana benthamiana* leaves (*35S:MdZFP3‐nLuc and 35S:MdHDA19‐cLuc*) using previously above methods. Luciferase activity was assessed as above described. To investigate the effect of *MdEAEL1* on the interaction between MdZFP3 and MdTPL4, the CDS of *MdEAEL1* was cloned into pRI101 to construct the *35S:MdEAEL1* plasmid. Following above method, the *35S:MdEAEL1* was transiently overexpressed in *N. benthamiana leaves* (*35S:MdZFP3‐cLuc* and *35S:MdTPL4‐nLuc*), which were treated with 50 µm of MG132 12 h prior to LUC imaging, as described above.

### Y1H Assay

Y1H assays were conducted to confirm the binding of MdZFP3 to the promoters of *MdPG1*, *MdPL5*, *Mdβ‐Gal9*, *Mdα‐AFase2*, *MdXET1*, and *MdEXP8*, and *MdEAEL1*. The CDS of *MdZFP3* was inserted into the pGADT7 vector (Clontech, USA), while promoter fragments of *MdPG1*, *MdPL5*, *Mdβ‐Gal9*, *Mdα‐AFase2*, *MdXET1*, and *MdEXP8*, and *MdEAEL1* (1200 bp upstream of the translation start site) were cloned into the pAbAi. The Y1H assay was performed as previously described.^[^ ^38^ ^]^

### Transient Expression Assays in *N. benthamiana* Leaves

Transient expression assays were performed as previously described.^[^ ^39^ ^]^ The CDSs of *MdZFP3*, *MdZFP3mEAR*, or *MdZFP3△EAR* were cloned into the pGreenII 62SK vector, which contains the GAL4 binding domain and VP16, generating the plasmid *pBD‐MdZFP3‐VP16*, *pBD‐MdZFP3mEAR*, or *pBD‐MdZFP3△EAR* for use as effectors. The *GAL4:LUC* construct contains five copies of the GAL4‐binding element driving the expression of *LUC*, as well as an internal control, *REN*, driven by the 35S promoter as a reporter. In addition, the CDSs of *MdZFP3*, *MdZFP3mEAR*, or *MdZFP3△EAR* were cloned into the pRI101 vector, generating the plasmids *35S:MdZFP3*, *35S:MdZFP3mEAR*, or *35S:MdZFP3△EAR* for use as effector factors. The *mini35S:LUC* construct contains five copies of the ZFP transcription factor binding element (AAGCCAC) driving the expression of *LUC*, as well as an internal control *REN* driven by the 35S promoter as a reporter. The effector and reporter constructs were co‐introduced into the *N. benthamiana* leaves. After 36 h of transfection, LUC and REN activities were assessed using a dual luciferase assay kit (Cat. no. E1910, Promega, Madison, WI, USA). The relative activity of LUC was determined by calculating the ratio of LUC to REN activity, as previously described.^[^ ^14^ ^]^

### GUS Assay

The *ProMdPG1:GUS*, *ProMdPL5:GUS*, *ProMdβ‐Gal9:GUS*, *ProMdα‐AFase2:GUS*, *ProMdXET1:GUS*, *ProMdEXP8:GUS*, or *ProMdEAEL1:GUS* reporter plasmids were constructed by inserting the *MdPG1*, *MdPL5*, *Mdβ‐Gal9*, *Mdα‐AFase2*, *MdXET1*, *MdEXP8*, or *MdEAEL1* promoter (1200 bp upstream of the ATG start codon) into the pCAMBIA1300‐GUS or pCAMBIA1300‐35Smini‐GUS vector. The CDSs of *MdZFP3*, *MdZFP3mEAR*, *MdZFP3△EAR*, *MdEAEL1*, *MdTPL4*, or *MdHDA19* were separately cloned into the pRI101 vector containing the 35S promoter to generate effector constructs. The reporter and effector plasmids were transformed into *N. benthamiana* leaves by Agrobacterium‐mediated infiltration and the relative GUS activity was calculated as previously described.^[^ ^7^ ^]^ The MG132 (50 µm) treatment was performed 12 h before detection.

### Dual‐Luciferase Reporter Assay

The *ProMdPG1:LUC*, *ProMdPL5:LUC*, *ProMdβ‐Gal9:LUC*, *ProMdα‐AFase2:LUC*, *ProMdXET1:LUC*, *ProMdEXP8:LUC*, or *ProMdEAEL1:LUC* reporter plasmids were created by inserting the MdPG1, MdPL5, Mdβ‐Gal9, Mdα‐AFase2, MdXET1, MdEXP8, or MdEAEL1 promoters (1200 bp upstream of the ATG start codon) into the pGreen II 0800‐Luc or pGreen II 0800‐35 Smini‐Luc vector. Effector constructs were generated by cloning the CDSs of MdZFP3, MdTPL4, MdHDA19, or MdEAEL1 into the pRI101 vector containing the 35S promoter. The reporter and effector plasmids were transformed into *N. benthamiana* leaves, and luciferase activity was measured as previously described.^[^ ^7^ ^]^ The MG132 (50 µm) treatment was performed 12 h before detection.

### ChIP‐qPCR Assay

To determine whether MdZFP3 binds to the promoters of *MdPG1*, *MdPL5*, *Mdβ‐Gal9*, *Mdα‐AFase2*, *MdXET1*, *MdEXP8*, or *MdEAEL1*, the *MdZFP3* CDS was inserted into the pRI101 vector downstream of the sequence encoding a GFP tag and a 35S promoter to generate the *35S:GFP‐MdZFP3* plasmid. The *35S:GFP‐MdZFP3* plasmid was then transformed into apple calli as described above. pRI101‐GFP (*35S:GFP*) transgenic apple calli were used as controls. To investigate the histone acetylation levels of the promoters of *MdPG1*, *MdPL5*, *Mdβ‐Gal9*, *Mdα‐AFase2*, *MdXET1*, *MdEXP8*, and *MdEAEL1*, fruit with transient overexpression (*MdZFP3*‐OE) or transient silencing (*MdZFP3*‐AS) of *MdZFP3* and fruit with transient overexpression (*MdEAEL1*‐OE) or transient silencing (*MdEAEL1*‐AS) of *MdEAEL1* were used as experimental samples, while fruit with transient expression of an empty vector served as the control. Subsequently, ChIP‐qPCR assays were conducted as previously described,^[^ ^38^ ^]^ using anti‐GFP, anti‐H3K9Ac (Cat. no. 9649, Cell Signaling Technologies), or anti‐H3K27Ac (Cat. no. 8137, Cell Signaling Technologies) antibody. The amount of immunoprecipitated chromatin was measured using quantitative PCR (qPCR) following a previously described method.^[^ ^7^ ^]^

### In Vitro and In Vivo Ubiquitination Assays

To produce and purify MdZFP3‐His and MdEAEL1‐GST proteins, the CDS of MdZFP3 was inserted into the pEASY‐E1 vector (Cat. no. CE111; Transgen Biotech), to generate the 6 × histidine (His) fusion proteins. Similarly, the CDS of MdEAEL1 was cloned into the pGEX‐4T‐1 vector (Cat. no. CW2198; CWBIO) to generate glutathione S‐transferase (GST) fusion proteins. These recombinant plasmids were separately transformed into *Escherichia coli* BL21 (DE3) cells, as previously described.^[^ ^36^ ^]^ The temperature used for MdZFP3‐His and MdEAEL1‐GST production was 16 °C and the final isopropylthio‐β‐galactoside (IPTG) concentration used to induce protein production was 0.5 mm. The protein purification protocol was as previously described.^[^ ^7^ ^]^ The in vitro ubiquitination assays were performed as previously reported.^[^ ^31^ ^]^ In summary, 50 ng of human E1, 50 ng of human E2, 200 ng of E3 (MdEAEL1), 10 µg of His‐6‐ubiquitin, and 100 ng of MdZFP3 were incubated in 30 µL of ubiquitination reaction buffer (50 mm Tris‐HCl pH 7.5, 10 mm MgCl_2_, 10 mm ATP, 1 mm DTT) at 30 °C for 4 h. The proteins were then fractionated by SDS‐PAGE, and the presence of ubiquitinated MdZFP3 was determined using an anti‐ubiquitin (Cat. no. ST1200, Sigma) or an anti‐His antibody. The in vivo ubiquitination assays were conducted as previously described.^[^ ^31^ ^]^ Briefly, apple fruit (Wild type, *MdEAEL1*‐OE, or *MdEAEL1*‐AS) and transgenic calli expressing *35S:Myc‐MdZFP3* or *35S:Myc‐MdZFP3* + *35S:MdEAEL1‐OE* were treated with 50 µm MG132 for 12 h. MdZFP3 was immunoprecipitated using an anti‐Myc or anti‐MdZFP3 antibody and the eluted proteins were analyzed by immunoblotting with the anti‐ubiquitin antibody (Cat. no. ST1200, Sigma, USA) as previously described.^[^ ^31^ ^]^

### Protein Degradation Assay

For the cell‐free degradation assay, MdZFP3‐His was expressed and purified as described above. Proteins were extracted from *35S:MdEAEL* transgenic apple calli using a degradation buffer (25 mm Tris‐HCl, pH 7.5, 10 mm NaCl, 10 mm MgCl_2_, 4 mm PMSF, 5 mm DTT, and 10 mm ATP), following the previously described.^[^ ^7^ ^]^ Wild type (Wt) served as the control. Recombinant MdZFP3‐His protein (10 µg) was added to the total protein extracts (500 µg), which contained either MG132 (50 µm) or dimethyl sulfoxide (DMSO), and incubated for 0, 3, or 6 h at 25 °C. The samples were then fractionated using SDS‐PAGE and MdZFP3‐His detected with an anti‐His antibody. For the LUC report gene method, the *MdZFP3* CDS was cloned into the pRI101‐LUC vector containing the 35S promoter to generate the construct *35S:MdZFP3‐LUC*. The *MdEAEL1* CDS was cloned into the pRI101 vector containing the 35S promoter to generate *35S:MdEAEL1*. The *Agrobacterium*‐mediated infiltration method was used to introduce *35S:MdZFP3‐LUC* alone or together with *35S:MdEAEL1* into *N. benthamiana* leaves and luciferase activity was measured as previously described.^[^ ^7^ ^]^ MG132 (50 µm) treatment was performed 12 h before detection.

### DNA Pull‐Down Assay

To produce and purify MdTPL4‐His, MdZFP3‐GST, and MdHDA19‐MBP proteins, the *MdTPL4* CDS was inserted into the pEASY‐E1 vector (Cat. no. CE111; Transgen Biotech) to generate the corresponding 6 × His fusion protein. The MdZFP3 CDS was cloned into the pGEX‐4T‐1 vector (Cat. no. CW2198; CWBIO) to generate the corresponding glutathione GST fusion protein. The MdHDA19 was introduced into the pMAL‐c2x vector^[^ ^7^ ^]^ to produce a MBP fusion protein. These recombinant plasmids were separately transferred into *E. coli* BL21 (DE3) cells to produce the target proteins, as previously described.^[^ ^36^ ^]^ The production temperature for MdTPL4‐His and MdZFP3‐GST was 16 and 23 °C MdHDA19‐MBP. The IPTG concentration for protein induction was 0.5 mm. The proteins were purified as previously described.^[^ ^7^ ^]^ The *MdPG1* promoter fragment was amplified by PCR using 5′‐biotin‐labeled primers. Following the incubation of MdTPL4‐His (50 µg) and MdHDA19‐MBP (50 µg) with biotin‐labeled DNA and either GST (40 µg) or MdZFP3‐GST (40 µg) protein, the DNA‐binding proteins were pulled down using streptavidin agarose beads. Immunoblotting was then performed using anti‐His, anti‐MBP, and anti‐GST antibodies, as previously described.^[^ ^14^ ^]^

### *Agrobacterium*‐Mediated Transformation of Apple Fruit

To transiently overexpress *MdZFP3*, *MdEAEL1*, *MdTPL4*, and *MdHDA19* in apples, *Agrobacterium*‐mediated transformation was performed as previously described.^[^ ^7^ ^]^ The CDS of *MdZFP3*, *MdEAEL1*, *MdTPL4*, or *MdHDA19* was inserted into the pRI101 vector to generate overexpression constructs (*35S:Myc‐MdZFP3*, *35S:Myc‐MdEAEL1*, *35S:MdTPL4*, and *35S:MdHDA19*). To silence *MdZFP3*, *MdEAEL1*, *MdTPL4*, and *MdHDA19* expression in apples, partial CDS fragments of *MdEAEL1* (87–216 bp), *MdZFP3* (141–624 bp), *MdTPL4* (225–845 bp), and *MdHDA19* (209–766 bp) were inserted into the pRI101 vector in the reverse direction to generate antisense silencing constructs (*35S:MdEAEL1‐AS*, *35S:MdZFP3‐AS*, *35S:MdTPL4*, and *35S:MdHDA19*). Each plasmid was introduced into *Agrobacterium* strain EHA105 using the heat‐shock method,^[^ ^40^ ^]^ with the pRI101‐Myc empty vectors as a control. Solutions for silencing or overexpression in apple fruits were prepared as previously described.^[^ ^33^ ^]^ One hundred microliters of *Agrobacterium* cell suspension containing the above plasmids was collected with a sterile 1 mL syringe and injected ≈0.5 cm deep into fruit 7 d before commercial harvest time.

### Statistical Analysis

Two‐tailed Student's *t*‐tests were performed on two sets of data using GraphPad Prism version 9.5. Asterisks denote significant differences between the two groups (*p < 0.05, **p < 0.01), while n.s. (*p* > 0.05) indicates no significant difference.

### Accession Numbers

The sequence data generated in this research are available at NCBI's GenBank (https://www.ncbi.nlm.nih.gov/), and GDR (https://www.rosaceae.org/) with the assigned accession numbers, including Mdactin (EB136338), *MdEAEL1* (MDP0000612469), *MdZFP3* (MDP0000183099), *MdTPL4* (LOC103433893), MdHDA19 (LOC103412136), *MdPG1* (MDP0000845685), *Mdβ‐Gal9* (MDP0000416548), *MdPL5* (MDP0000277149), *MdXET1* (MDP0000398765), *Mdα‐aFase2* (MDP0000140483), and *MdEXP8* (MDP0000431696).

## Conflict of Interest

The authors declare no conflict of interest.

## Author Contributions

T.L. and A.W. conceived and designed the study. T.L. performed most of the experiments. L.L., Y.C., and G.Y. performed gene expression analyses. B.S., L.S., and W.L. performed protein extraction. Y.W. cultivated and treated the apples. T.L. wrote the manuscript. All authors analyzed the data and discussed the article.

## Supporting information



Supporting Information

Supplemental Table 1

Supplemental Table 2

Supplemental Table 3

## Data Availability

Research data are not shared.
