# Supplementary material for: Ethylene‐Activated E3 Ubiquitin Ligase MdEAEL1 Promotes Apple Fruit Softening by Facilitating the Dissociation of Transcriptional Repressor Complexes
Source: Adv Sci (Weinh). 2025 Apr 9;12(22):2417393. doi: 10.1002/advs.202417393 (PMC12165066; doi:10.1002/advs.202417393)
Supplement: Supplementary file 1 — Supporting Information [file ADVS-12-2417393-s001.docx]

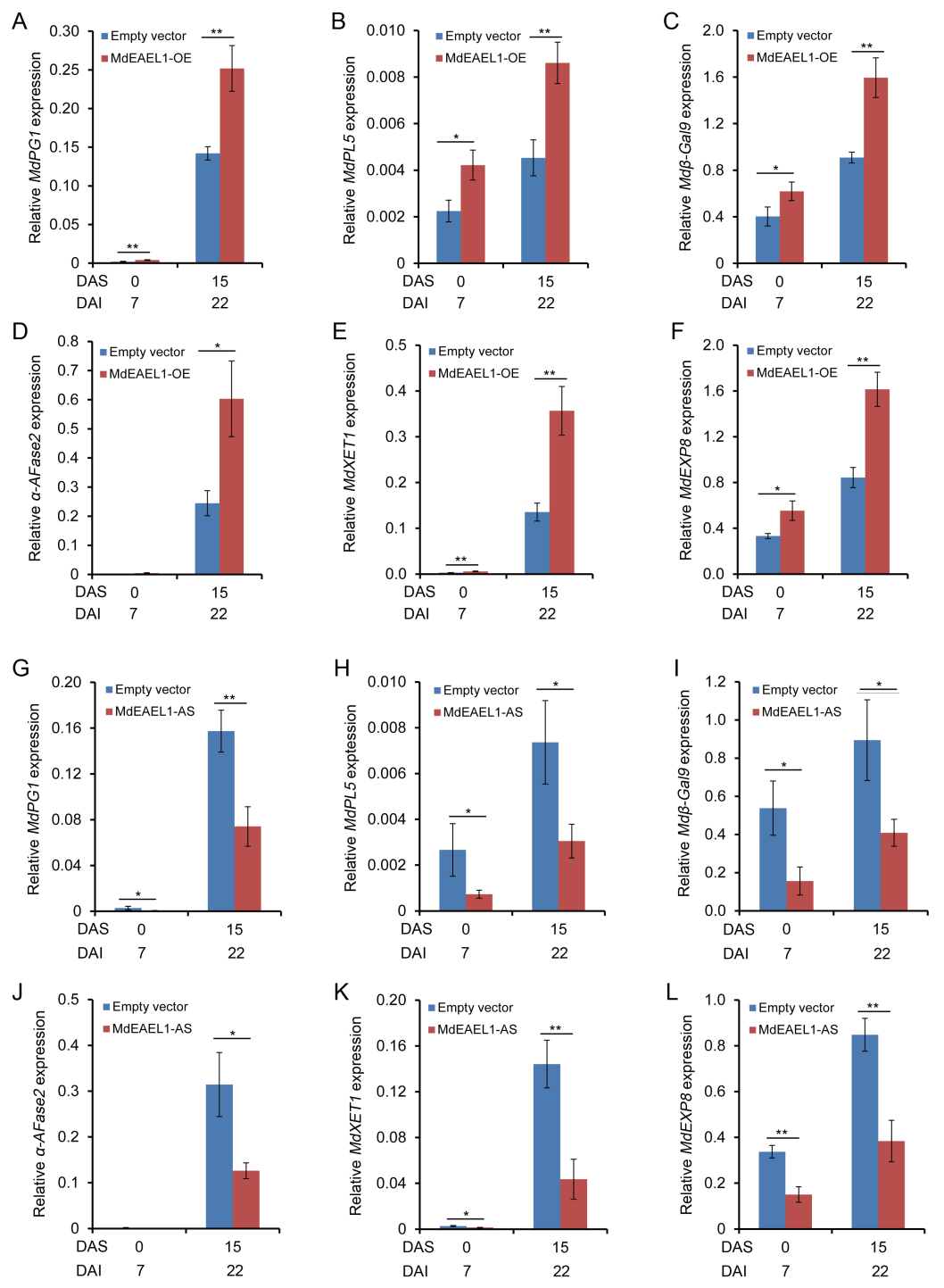


**Supplemental Figure S1. Expression of cell wall degradation-related genes in *MdEAEL1*-OE and *MdEAEL1*-AS fruit.**

**A-F)** Expression levels of *MdPG1* (A), *MdPL5* (B), *Mdβ-Gal9* (C), *Mdα-AFase2* (D), *MdXET1* (E), and *MdEXP8* (F) were detected by reverse transcription-quantitative PCR (RT-qPCR) in transiently overexpressing *MdEAEL1* (*MdEAEL1*-OE) apple fruit. Transient expression with the pRI101 plasmid (Empty vector) as a control. **G-L)** Expression levels of *MdPG1* (G), *MdPL5* (H), *Mdβ-Gal9* (I), *Mdα-AFase2* (J), *MdXET1* (K), and *MdEXP8* (L) were detected by RT-qPCR in transiently silenced *MdEAEL1* (*MdEAEL1*-AS) apple fruit. Transiently pRI101 plasmid (Empty vector) was used as a control. The data statistical analysis was used as described in Fig. 2.


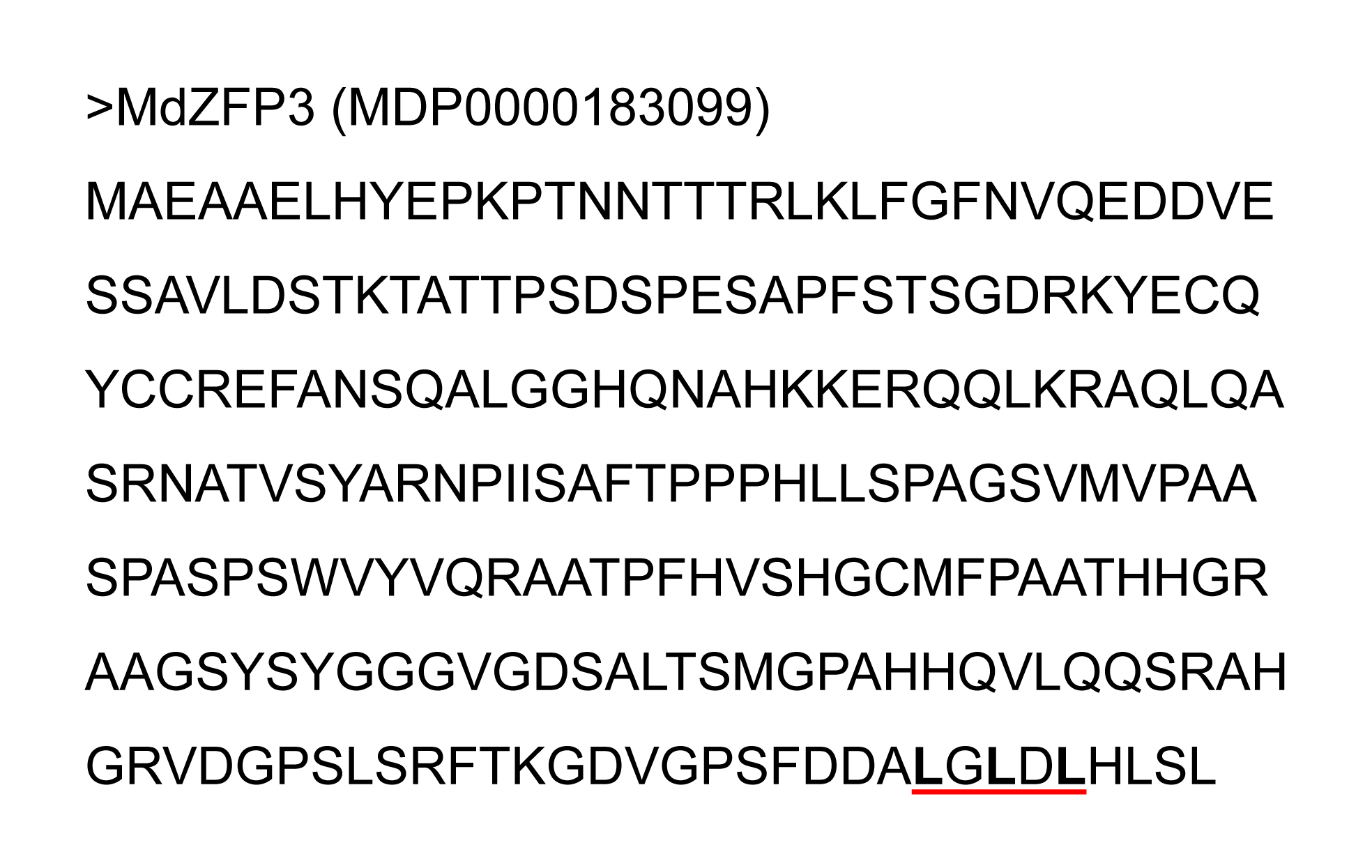


**Supplemental Figure S2. The protein sequence of MdZFP3 contains an EAR motif.**

The position marked with a red underline is the EAR motif.


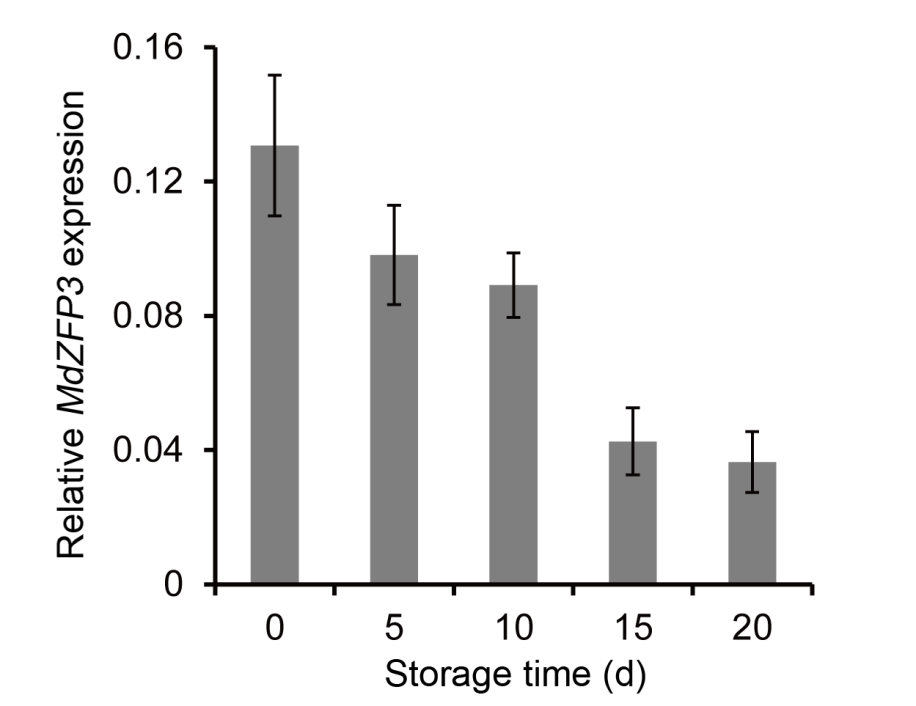


**Supplemental Figure S3. Expression of *MdZFP3* during apple fruit storage.**

Expression levels of *MdZFP3* was detected by reverse transcription-quantitative PCR (RT-qPCR). The data are presented as means ± SE (*n*=3 groups, 10 fruits per group).


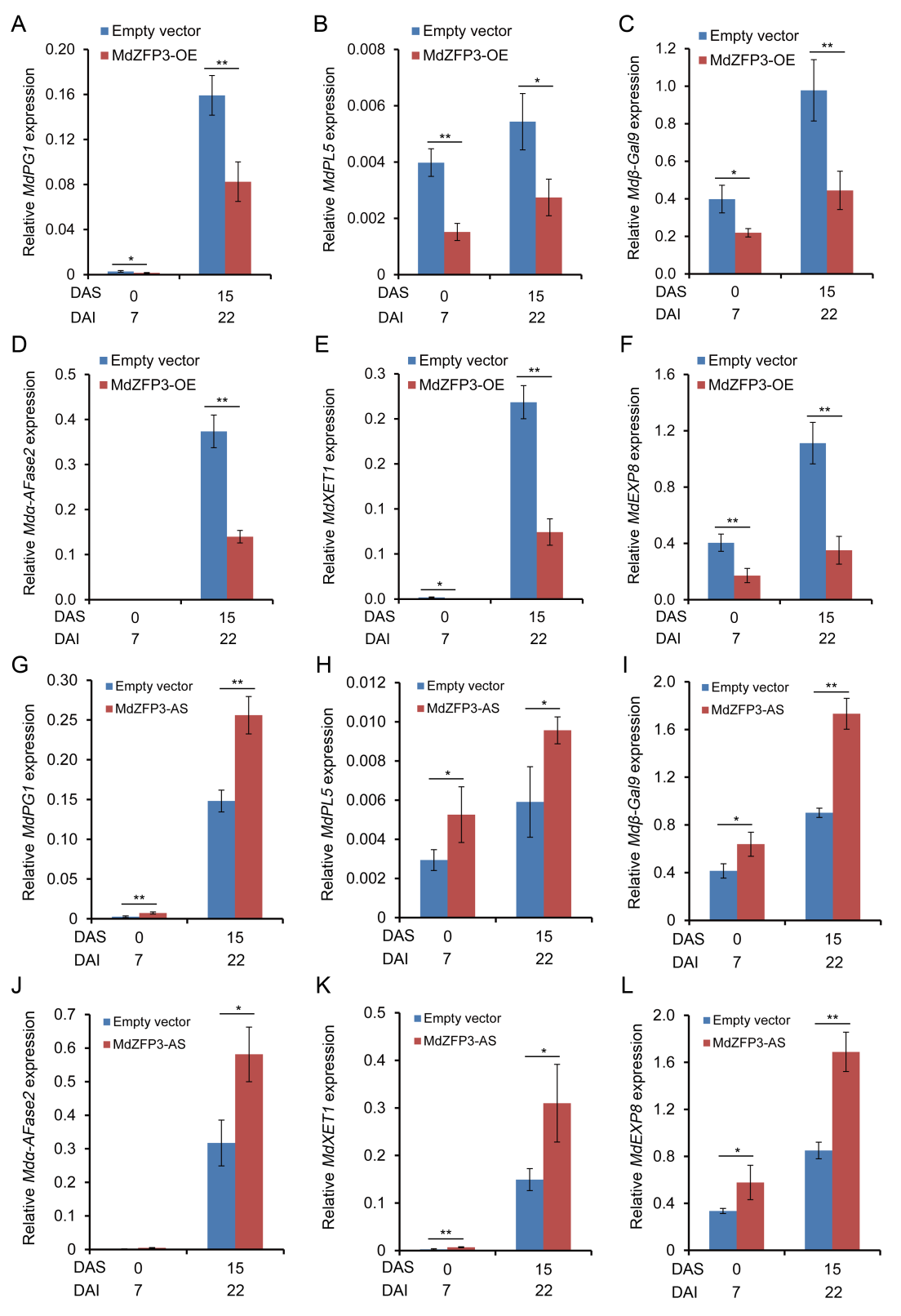


**Supplemental Figure S4. Expression of cell wall degradation-related genes in *MdZFP3*-OE and *MdZFP3*-AS fruit.**

Expression levels of *MdPG1* (A), *MdPL5* (B), *Mdβ-Gal9* (C), *Mdα-AFase2* (D), *MdXET1* (E), and *MdEXP8* (F) were detected by reverse transcription-quantitative PCR (RT-qPCR) in transiently overexpressing *MdZFP3* (*MdZFP3*-OE) apple fruit. Transiently pRI101 plasmid (Empty vector) was used as a control. **G-L)** Expression levels of *MdPG1* (G), *MdPL5* (H), *Mdβ-Gal9* (I), *Mdα-AFase2* (J), *MdXET1* (K), and *MdEXP8* (L) were detected by RT-qPCR in transiently silencing *MdZFP3* (*MdZFP3*-AS) apple fruit. Transiently pRI101 plasmid (Empty vector) was used as a control. The data statistical analysis was used as described in Fig. 2.


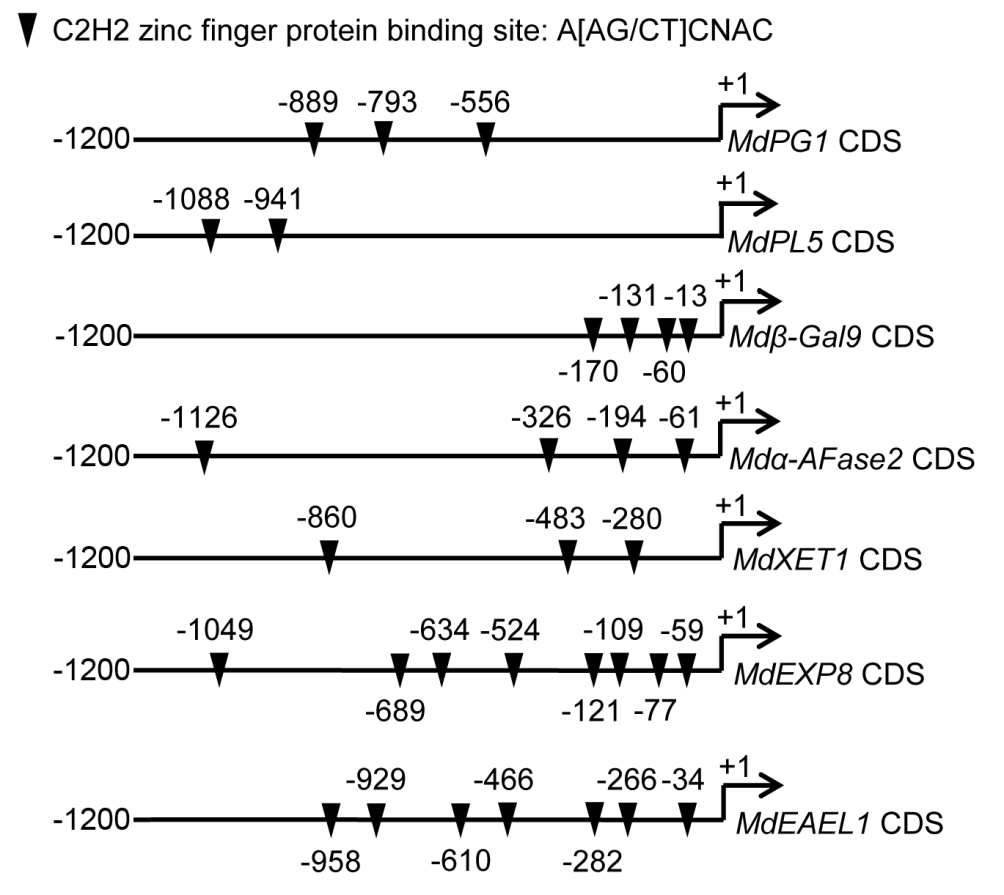


**Supplemental Figure S5. Binding sites of C2H2-type transcription factors in the promoters of cell wall degradation-related genes and MdEAEL1.**

**
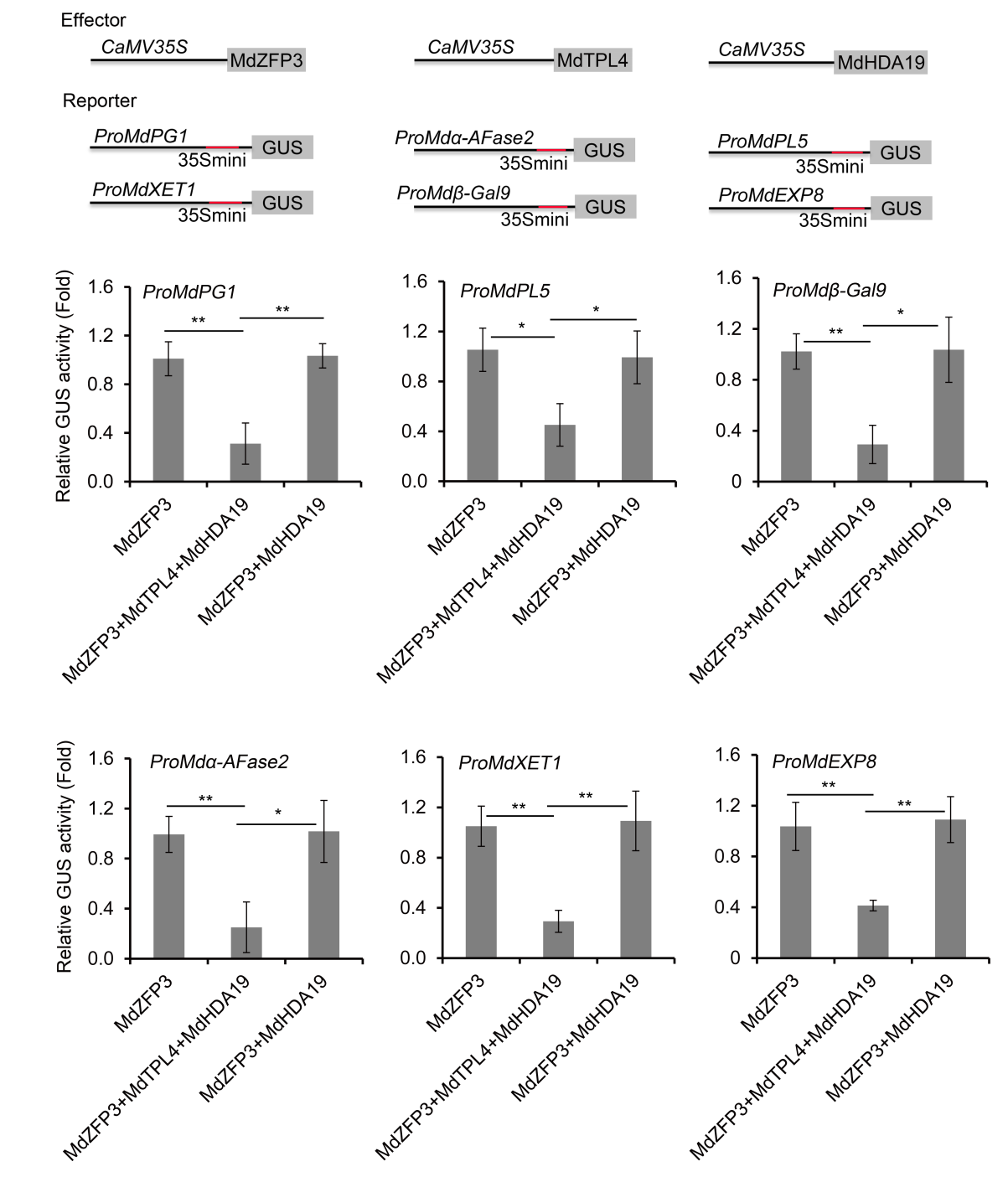
**

**Supplemental Figure S6. MdZFP3-MdTPL4-MdHDA19 transcriptional repression complex inhibits the expression of cell wall degradation-related genes.**

The GUS reporter plasmid was co-transfected into *N. benthamiana* leaves together with individual effector plasmids. The data are presented as means ± SE (*n*=3 independent transfected *Nicotiana benthamiana* leaves). Statistical significance was determined using Student’s *t*-test (***P* < 0.01, **P* < 0.05).


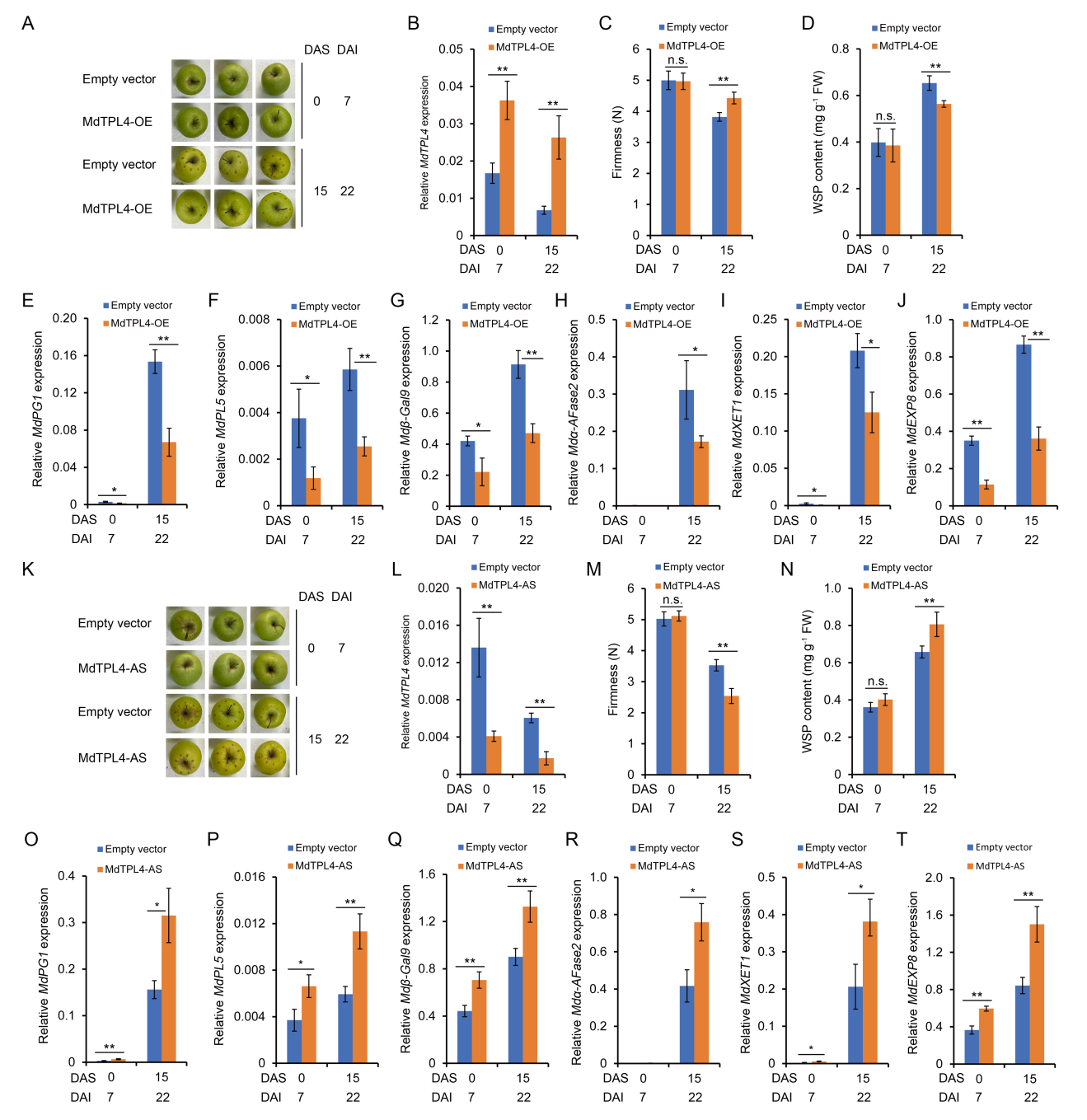


**Supplemental Figure S7. *MdTPL4* inhibits apple fruit softening during storage.**

**A)** Apple fruit transiently overexpressing *MdTPL4* (*MdTPL4*-OE) or empty vector (pRI101) during storage. *MdTPL4*-OE fruit were harvested 7 d after injection and stored at room temperature for 15 d. **B)** Reverse transcription-quantitative PCR (RT-qPCR) was used to detect the expression of *MdTPL4* in the fruits of *MdTPL4*-OE and Empty vector. **C and D)** Fruit firmness (C) and water-soluble pectin (WSP) (D) were measured. FW, Fresh weight. **E-J)** Expression levels of *MdPG1* (E), *MdPL5* (F), *Mdβ-Gal9* (G), *Mdα-AFase2* (H), *MdXET1* (I), and *MdEXP8* (J) were detected by RT-qPCR in transiently overexpressing *MdTPL4* (*MdTPL4*-OE) apple fruit. Transiently pRI101 plasmid (Empty vector) was used as a control. **K)** Apple fruit with transiently silenced *MdTPL4* expression (*MdTPL4*-AS), with an empty vector as a control. **L)** *MdTPL4*-AS fruit were harvested 7 d after injection and stored at room temperature for 15 d. RT-qPCR analysis of the expression of *MdTPL4* in the fruits of *MdTPL4*-AS and Empty vector. **M and N)** Fruit firmness (M) and water-soluble pectin (WSP) (N) were measured. FW, Fresh weight. **O-T)** Expression levels of *MdPG1* (O), *MdPL5* (P), *Mdβ-Gal9* (Q), *Mdα-AFase2* (R), *MdXET1* (S), and *MdEXP8* (T) were detected by RT-qPCR in transiently silencing *MdTPL4* (*MdTPL4*-AS) apple fruit. Transiently pRI101 plasmid (Empty vector) was used as a control. DAI, days after infiltration; DAS, days after storage. For firmness and WSP determination, as well as RT-qPCR, the data statistical analysis was used as described in Fig. 2.


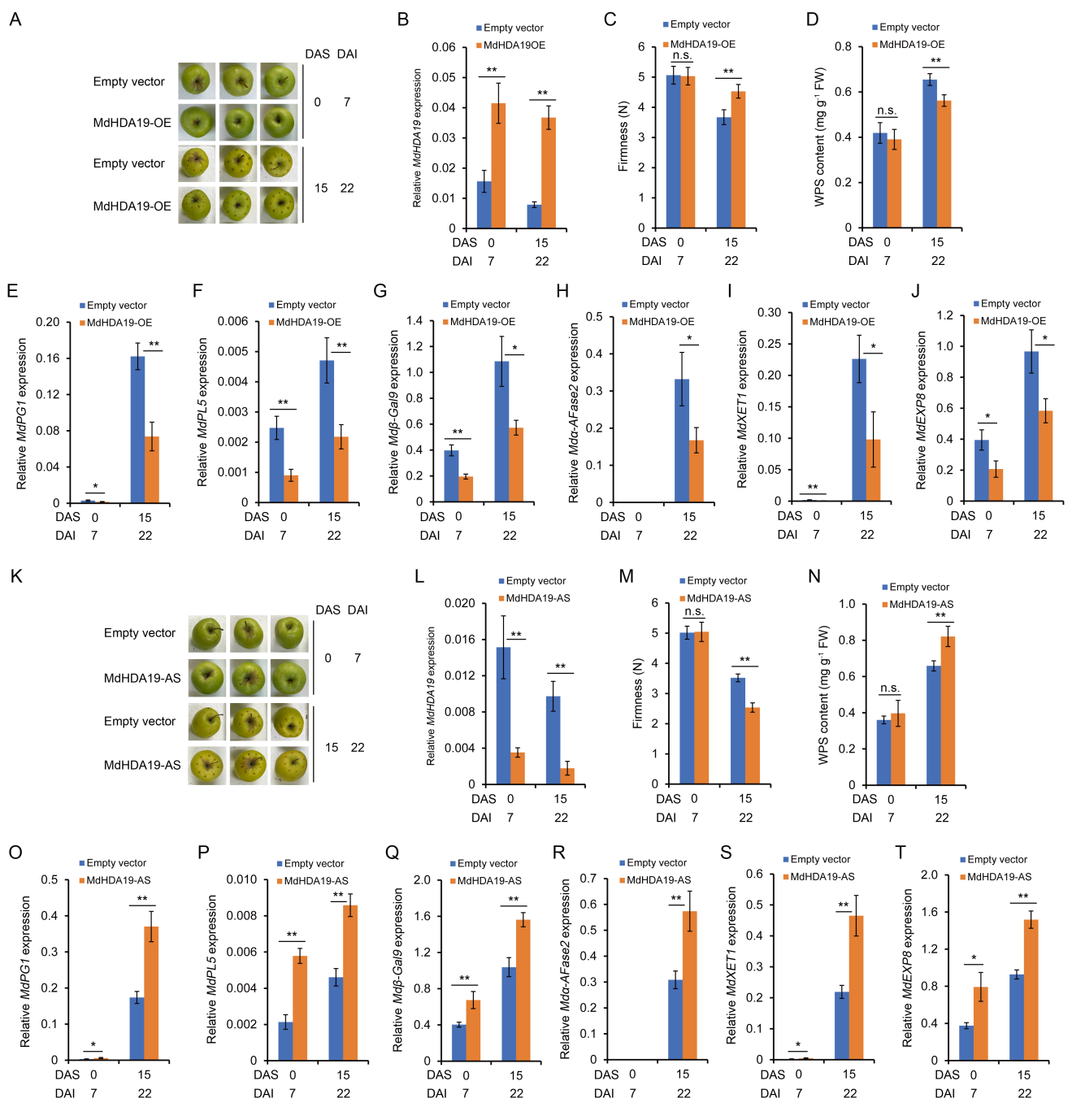


**Supplemental Figure S8. *MdHDA19* inhibits apple fruit softening during storage.**

**A)** Apple fruit transiently overexpressing *MdHDA19* (*MdHDA19*-OE) or empty vector (pRI101) during storage. *MdHDA19*-OE fruit were harvested 7 d after injection and stored at room temperature for 15 d. **B)** Reverse transcription-quantitative PCR (RT-qPCR) was used to detect the expression of *MdHDA19* in the fruits of *MdHDA19*-OE and Empty vector. **C and D)** Fruit firmness (C) and water-soluble pectin (WSP) (D) were measured. FW, Fresh weight. **E-J)** Expression levels of *MdPG1* (E), *MdPL5* (F), *Mdβ-Gal9* (G), *Mdα-AFase2* (H), *MdXET1* (I), and *MdEXP8* (J) were detected by RT-qPCR in transiently overexpressing *MdHDA19* (*MdHDA19*-OE) apple fruit. Transiently pRI101 plasmid (Empty vector) was used as a control. **K)** Apple fruit with transiently silenced *MdHDA19* expression (*MdHDA19*-AS), with an empty vector as a control. **L)** *MdHDA19*-AS fruit were harvested 7 d after injection and stored at room temperature for 15 d. RT-qPCR analysis of the expression of *MdHDA19*in the fruits of *MdHDA19*-AS and Empty vector. **M and N)** Fruit firmness (M) and water-soluble pectin (WSP) (N) were measured. FW, Fresh weight. **O-T)** Expression levels of *MdPG1* (O), *MdPL5* (P), *Mdβ-Gal9* (Q), *Mdα-AFase2* (R), *MdXET1* (S), and *MdEXP8* (T) were detected by RT-qPCR in transiently silencing *MdTHDA19* (*MdHDA19*-AS) apple fruit. Transiently pRI101 plasmid (Empty vector) was used as a control. DAI, days after infiltration; DAS, days after storage. For firmness and WSP determination, as well as RT-qPCR, the data statistical analysis was used as described in Fig. 2.


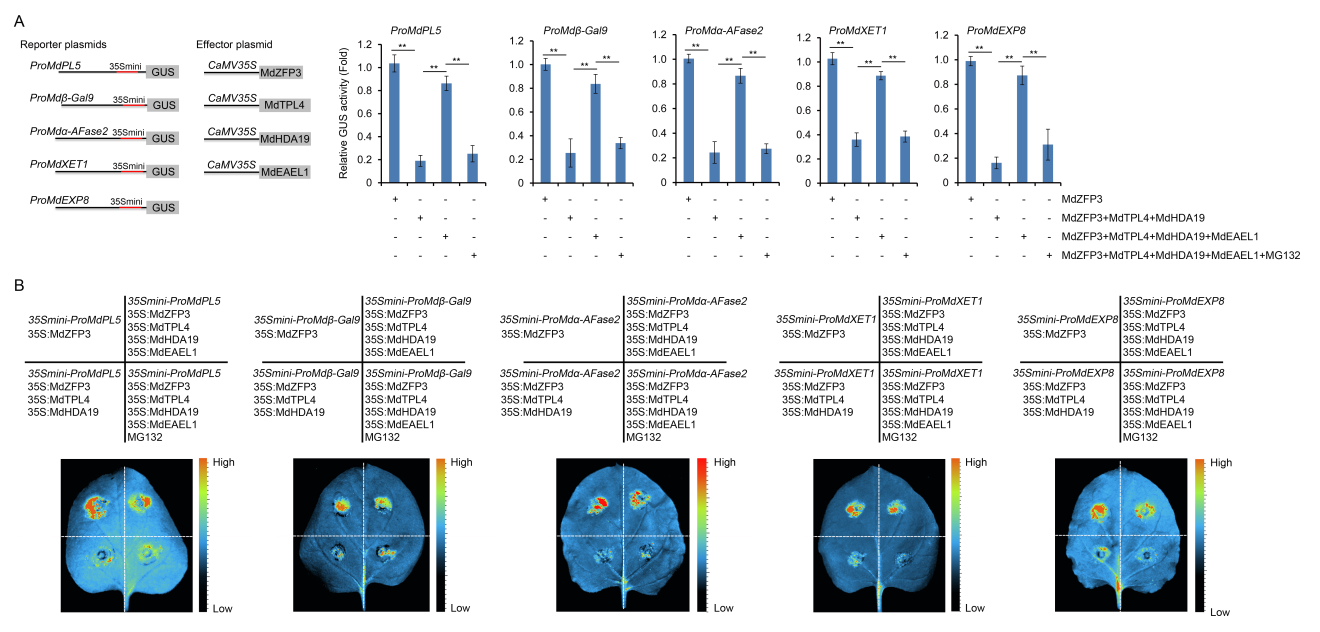


**Supplemental Figure S9. MdEAEL1 mediates the disassembly of the MdZFP3-MdTPL4-MdHDA19 complex, promoting the transcription of cell wall degradation-related genes.**

**A)** GUS reporter assays indicating that MdEAEL1 mediates the disassembly of the MdZFP3-MdTPL4-MdHDA19 complex, promoting the transcription of cell wall degradation-related genes. The GUS reporter plasmid was co-transfected into *N. benthamiana* leaf together with individual effector plasmids. **B)** The LUC reporter was co-transfected into *N. benthamiana* leaves together with individual effector plasmids.


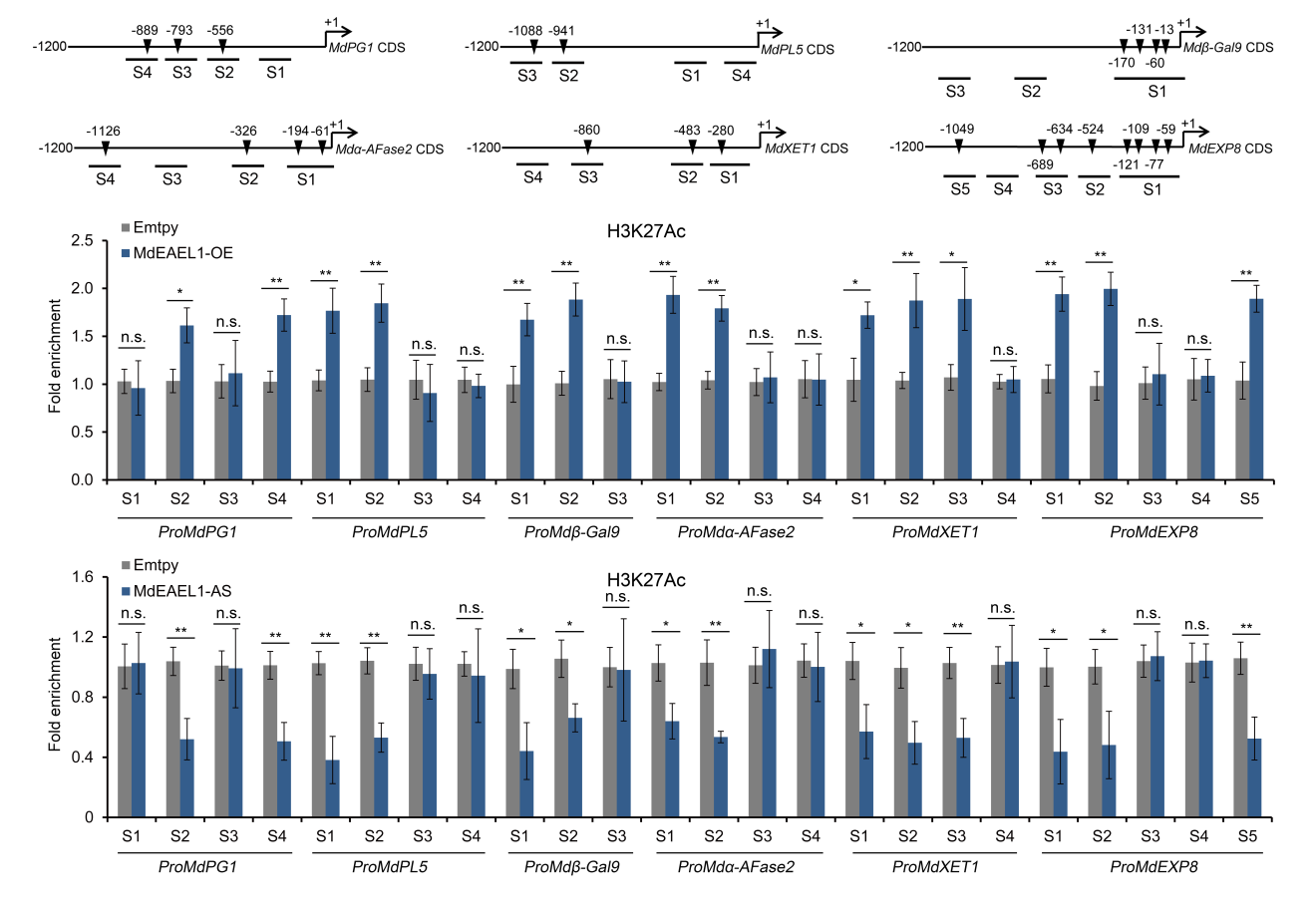


**Supplemental Figure S10. *MdEAEL1* upregulates histone acetylation in the promoter region of MdZFP3 target genes.**

Chromatin immunoprecipitation (ChIP)-qPCR analysis of H3K27Ac levels in the *MdPG1*, *MdPL5*, *Mdβ-Gal9*, *Mdα-AFase2*, *MdXET1*, and *MdEXP8* promoters in *MdEAEL1*-OE and *MdEAEL1*-AS fruit at 15 d after harvest. The empty vector transgenic fruit (Empty vector) were used as a control. The data statistical analysis was as described in Fig. 7B.


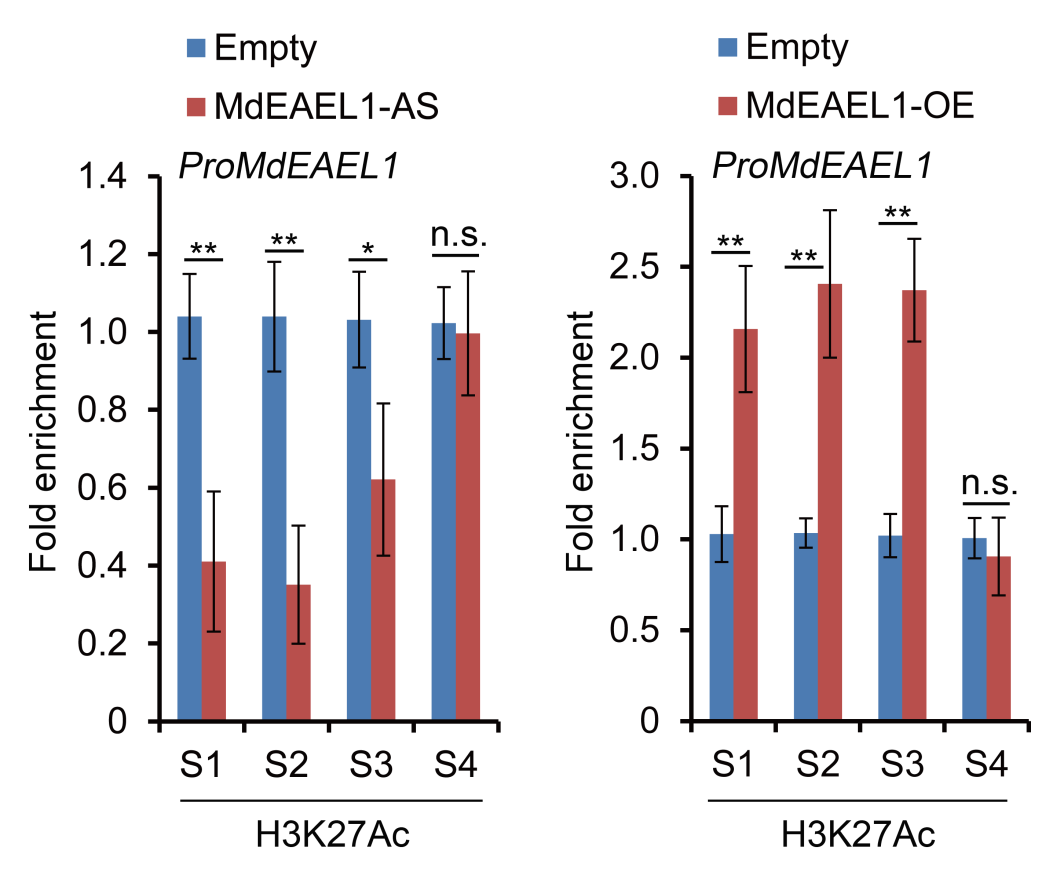


**Supplemental Figure S11. MdEAEL1 alters the histone acetylation level of its own promoter.**

Chromatin immunoprecipitation (ChIP)-qPCR analysis of H3K27Ac level at the *MdEAEL1* promoter in *MdEAEL1*-OE and *MdEAEL1*-AS fruit at 15 d after harvest. The empty vector transgenic fruit (Empty vector) were used as a control. The data statistical analysis was as described in Fig. 7B.
